# +Clonal stochasticity in early NK cell response to mouse cytomegalovirus is generated by mature subsets of varying proliferative ability

**DOI:** 10.7554/eLife.104951

**Published:** 2026-09-14

**Authors:** Darren Wethington, Saeed Ahmad, Marc Potempa, Giuseppe Giuliani, Oscar A Aguilar, Maheshwor Poudel, Simon Grassmann, William C Stewart, Nicholas M Adams, Joseph C Sun, Lewis L Lanier, Jayajit Das

**Affiliations:** 1 Steve and Cindy Rasmussen Institute for Genomic Medicine, Nationwide Children’s Hospital Columbus United States; 2 Biomedical Sciences Graduate Program, The Ohio State University Columbus United States; 3 Department of Microbiology and Immunology and the Parker Institute for Cancer Immunotherapy, University of California, San Francisco San Francisco United States; 4 Department of Physics, The Ohio State University Columbus United States; 5 Immunology Program, Memorial Sloan Kettering Cancer Center New York United States; 6 GIG Statistical Consulting, LLC Columbus United States; 7 Department of Pathology, New York University Grossman School of Medicine New York United States; 8 Department of Pediatrics, The Ohio State University Columbus United States; 9 Pelotonia Institute for Immuno-Oncology, College of Medicine, The Ohio State University Columbus United States; Friedrich-Alexander-University Erlangen-Nürnberg Germany; National Institute of Immunology India

**Keywords:** NK cell clonal expansion, memory NK cells, stochastic fluctuations, Master equation, clonal heterogeneity, MCMV infection, Mouse

## Abstract

Natural killer (NK) cells are classically defined as innate immune cells, but experiments show that mouse cytomegalovirus (MCMV) infection in C57BL/6 mice can cause NK cells to undergo antigen-specific proliferation and memory formation, similar to adaptive CD8+ T cells. One shared behavior between CD8+ T cells and NK cells is clonal expansion, where a single stimulated cell proliferates rapidly to form a diverse population of cells. For example, clones derived from single cells are most abundant during expansion when they are primarily CD27- for NK cells and CD62L- for T cells, phenotypes derived from precursor CD27+ and CD62L + cells, respectively. Here we determined the mechanistic rules involving proliferation, cell death, and differentiation of endogenous and adoptively transferred NK cells in the expansion phase of the response to MCMV infection. We found that the interplay between cell proliferation and cell death of mature CD27- NK cells and a highly proliferative CD27-Ly6C- mature subtype and intrinsic stochastic fluctuations in these processes play key roles in regulating the heterogeneity and population of the NK cell subtypes. Furthermore, we estimate rates for maturation of endogenous NK cells in homeostasis and in MCMV infection and found that only NK cell growth rates, and not differentiation rates, are appreciably increased by MCMV. Taken together, these results quantify the differences between the kinetics of NK cell antigen-specific expansion from that of CD8+T cells and unique mechanisms that give rise to the observed heterogeneity in NK cell clones generated from single NK cells in the expansion phase.

## Introduction

Natural killer (NK) cells are lymphocytes of the innate immune system and provide important protection against viral infections and tumors (Caligiuri, 2008; Vivier et al., 2008). NK cells develop in the bone marrow and express a diverse set of germline-encoded activating and inhibitory receptors (Abel et al., 2018; Biassoni, 2008; Huntington et al., 2007). Mouse cytomegalovirus (MCMV) infection induces antigen-specific expansion of naïve Ly49H+NK cells in mice, followed by a contraction phase, and then the formation of long-lived adaptive NK cells displaying recall responses to the same antigen (Cerwenka and Lanier, 2016; Robbins et al., 2004; Schlub et al., 2011; Sun et al., 2009). The activating Ly49H receptor, encoded by *Klra8*, recognizes the MCMV-encoded m157 glycoprotein and leads to activation of Ly49H+NK cells (Orr et al., 2009; Voigt et al., 2003). In humans, a similar activation of NK cells by human cytomegalovirus is observed with the preferential expansion of NKG2C+NK cells (Gumá et al., 2004; Hendricks et al., 2014; Ishiyama et al., 2022; Kim et al., 2019; Lopez-Vergès et al., 2011). The expansion, contraction, and memory formation of Ly49H+NK cells during MCMV infection bears similarity to antigen-specific responses of adaptive CD8+ T cells that generate memory CD8+ T cells (Adams et al., 2020; Buchholz et al., 2013; Kaech et al., 2002; Schlub et al., 2011).

A range of experiments investigated epigenetic changes, RNA transcription, and surface protein expression in single and bulk Ly49H+NK cells undergoing expansion and contraction in mice responding to MCMV infection (Flommersfeld et al., 2021; Grassmann et al., 2019; Lau et al., 2018; Martinet et al., 2015; Min-Oo et al., 2014; Nabekura et al., 2014; Potempa et al., 2022; Riggan et al., 2022). These observations show that the NK cell compartment undergoes phenotypic changes, resulting in changes in the abundances of varying subpopulations of cells. The development of these subpopulations depends on the three main signal inputs, antigen stimulation of a specific receptor (e.g., Ly49H), co-stimulation, and cytokine signaling (Ni et al., 2012; Peng and Tian, 2017; Romee et al., 2016). These subpopulations express distinct epigenetic, transcriptomic, and protein markers imprinted by signaling and gene regulatory processes. Observations of clonal expansion of barcoded single Ly49H+NK cells adoptively transferred into *Rag2^−/−^Il2rg^−/−^* mice revealed another layer of heterogeneity where sizes and compositions of NK cell clones originating from single naïve Ly49H+NK cells vary widely (Flommersfeld et al., 2021; Grassmann et al., 2019). This heterogeneity in the NK cell population is regulated by the stochastic kinetics of cell proliferation, cell death, and differentiation involving the subpopulations that emerge as the NK cells respond to the infection. Analysis of similar heterogeneities in clonal CD8+ T cell populations Buchholz et al., 2013; Gerlach et al., 2013 have shown generation of a highly proliferative short-lived effector phenotype and intrinsic stochastic fluctuations in the differentiation and proliferation processes, which account for heterogeneity of the CD8+ T cell clones (Buchholz et al., 2013). These effector CD8+ T cells are CD62L- and differentiate during the expansion phase from upstream memory precursors that are CD62L+ (Schlub et al., 2010; Yang et al., 2011). More recently, tracking proliferation, death, and differentiation of CD8+ T cells during clonal expansion has introduced the notion of division destiny where antigen and cytokine stimulation in an individual T cell determines the division destiny or the number of divisions the T cell undergoes before differentiating into a state with negligible proliferation (Cheon et al., 2021; De Boer and Yates, 2023; Hawkins et al., 2007; Heinzel et al., 2017). More generally, these studies demonstrated that the fates of individual T or B cells during clonal expansion are linked to the number of divisions executed by those individual cells (Bresser et al., 2022; De Boer and Yates, 2023). Modeling the expansion phase of CD8+ T cell clones incorporating division destiny, heterogeneous antigen stimulation times, inheritance of division and death times in daughter cells from mother T cell, and cell division-dependent kinetics of cell lineage marker (e.g., CD62L) expressions (Pandit and De Boer, 2019) showed that the model can qualitatively recapitulate the observed heterogeneity (Buchholz et al., 2013) in the CD8+ T cell population in the expansion phase. The above models known as Cyton models investigate stochastic kinetics of individual T/B cells during clonal expansion and contraction. The existence of division destiny in antigen-stimulated Ly49H+NK cells still needs to be elucidated (Rückert and Romagnani, 2024).

Despite the qualitative similarities between the antigen-specific responses of NK cells and CD8+ T cells there are several marked differences. Single-cell NK clones typically undergo a relatively smaller 1000× increase in number compared to the 40,000×increase in CD8+ T cell populations (Adams et al., 2020). Additionally, mature CD27- NK cells grow more slowly than their immature CD27+ counterparts (Buchholz et al., 2013; Hayakawa and Smyth, 2006; Paul et al., 2016), whereas effector CD62L- CD8+ T cells proliferate faster than precursor CD62L+subsets (Adams et al., 2020; Kretschmer et al., 2020). These data suggest there may be mechanistic differences in antigen-specific NK and CD8+ T cell responses.

Here, we addressed whether distinct mechanisms underlie the expansion of NK cells and CD8+ T cells during antigen-specific responses by performing stochastic population dynamic modeling on published bulk and single-cell NK cell data collected during the expansion phase following MCMV infection in C57BL/6 mice. Our analysis and modeling show that the mechanistic rules that describe the expansion of CD8+ T cells are inadequate for describing the development of MCMV-specific NK cells. We found that mature CD27-Ly6C- NK cells undergo a greater rate of cell proliferation compared to immature CD27+ NK cells and more mature CD27-Ly6C+NK cells, and mature CD27- NK cells undergo increased death relative to the CD27+ immature cells. The variations in the timescales of proliferation and death processes coupled with the intrinsic stochastic fluctuations within these processes among the NK cell subsets regulate the heterogeneity in the sizes of the NK cell clones originated from individual immature NK cells during MCMV infection. Quantification of the population kinetics of Ly49H+NK cells in MCMV infection in the expansion phase shows a 10-fold increase in the cell growth rate compared to that in homeostatic conditions, whereas the effect of the infection on the maturation rate appears to be negligible. We also find that growth rates of adoptive NK cells in transfer experiments during MCMV infection in the expansion phase are higher than those of endogenous NK cells.

## Results

### A minimal progressive two-stage immature to mature differentiation model cannot capture the heterogeneity of the NK cell clones in the expansion phase

**Figure 1.**
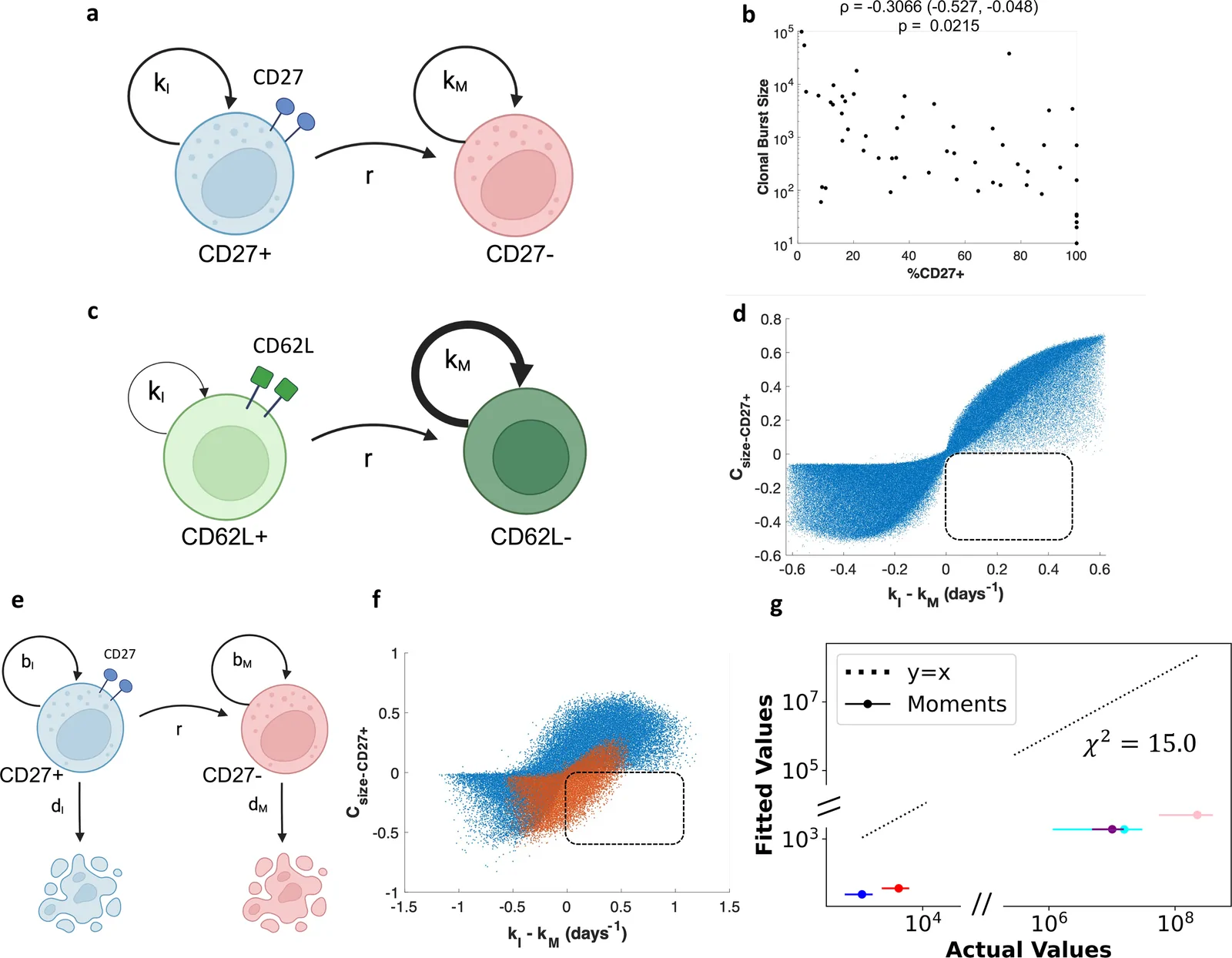
Two-stage linear models cannot capture desired properties of experimental clonal burst data. (**a**) Schematic representation of the two-stage model. The model describes immature (CD27+) cells and mature (CD27-) cells. Each cell type has a distinct growth rate (*k_I_* for immature, *k_M_* for mature), and immature cells differentiate to mature cells at rate *r*. (**b**) Experimental data from Flommersfeld et al. display negative value for the correlation *C_size-CD27+_* between clonal burst size and %CD27+ cells. (**c**) Schematic description of antigen-specific clonal proliferation and differentiation in CD8+ T cells. Because mature CD62L- cells proliferate more rapidly compared to the immature CD62L+ cells, initial clones made primarily of CD62L- cells can grow to generate the larger size clones. (**d**) A parameter scan of all possible combinations of *k_I_*, *k_M_*, and *r* shows that negative correlations (*C_size-CD27+_*<0) are not realized when *k_I_ > k_M_*. Each point represents a value of *C_size-CD27+_* obtained from the model at a unique value of set of model parameters. The parameter configurations that populate the bottom-right quadrant satisfy our two constraints imposed by experimental observations regarding the negative values of *C_size-CD27+_* and higher growth rate of immature NK cells compared to their mature counterparts. (**e**) Schematic representation of the three-stage model including cell death. The model has separate parameters for birth of immature (CD27+) cells (*b_I_*), death of immature cells (*d_I_*), birth of mature (CD27-) cells (*b_M_*), death of mature cells (*d_M_*), and differentiation of immature cells to mature (r). In this model, the growth rates are defined as *k_I_ = b_I_ d_I_* and *k_M_ = b_M_ d_M_*. These net rates relate to the rates of growth or loss of clonal populations. (**f**) A parameter scan for this model shows that some parameter configurations can meet the two constraints where *k_I_ > k_M_* and the parameters result in negative values for *C_size-CD27+_*. Each point represents a unique parameter configuration. Red points denote parameter configurations where *b_M_ > b_I_* and *d_M_ > d_I_*. (**g**) Shows the best fit of the model in (**e**) to the moments with the constraints *k_I_ > k_M_* and *C_size-CD27+_*<–0.2. Circles represent the fitted values of the first and second moments for the distribution of the NK cell clones (n = 55) obtained from the model. Blue and red circles represent mean populations of CD27+, and CD27- NK cells, respectively. The variances of the CD27+ and CD27- NK cells are shown in cyan and pink circles, and the covariance between the CD27+ and CD27- NK cells is shown in purple. Horizontal lines around the circles show the standard deviations of bootstrapped moments (10,000 bootstraps). The solid line is the line *y=x*, which demonstrates where values would lie if we had a perfect fit.

We began our investigations by studying whether a minimal two-stage progressive differentiation model (Figure 1a) could describe the size and composition of NK cell clones that originated from single immature Ly49H+CD27+NK cells at day 8 post-infection with MCMV, a time typically at the peak of expansion. The differentiation of immature CD27+ to mature CD27- NK cells during MCMV infection and in homeostasis has been widely documented and established in previous experiments (Chiossone et al., 2009; Grassmann et al., 2019; Min-Oo et al., 2014; Mitrović et al., 2012). We used data collected by Flommersfeld et al., 2021, in which they adoptively transferred single genetic marker barcoded Ly49H+NK cells to a host that is subsequently infected with MCMV. Splenocytes were then harvested, and resulting NK cell clones were distinguished with flow cytometry, and CD27+ and CD27- cells were determined with flow cytometry gates. We characterized the NK cell clones by the means and variances of the populations of CD27+ or CD27- NK cells and the co-variance between the two cell types in the clones. The NK cell clones reported by Flommersfeld et al. contained mixtures of CD27+ and CD27- NK cells in the spleen. We evaluated the percentage of CD27+ NK cells in each clone and computed the correlation (*C_size-CD27+_*) of the size of the clone with the percentage of CD27+ NK cells in the clones. The larger size NK cell clones contained a greater proportion of the CD27- NK cells and thus produced a negative correlation between the clone size and the percentage of CD27+ NK cells in individual clones (Figure 1b). This finding is in line with that found in a similar experiment by Grassmann et al., 2019. These authors also transferred congenically marked CD27- and CD27+ Ly49H+NK cells in a 1:1 ratio at day 0 and then assayed the expanded CD27+/-NK cells at day 8 post infection, showing a higher proportion of mature CD27- NK cells compared to the immature CD27+ NK cells (Figure S2E in Grassmann et al., 2019). This result is supported by other experiments (Hayakawa and Smyth, 2006; Paul et al., 2016), and we used this observation to constrain our model parameter space. Heterogeneity of clones of CD8+ T cells generated by antigenic SIINFEKL stimulation showed a similar pattern where the largest clones in the expansion phase (day 7 post-stimulation) are composed of higher fractions of the mature CD62L- CD8+ T cells (Buchholz et al., 2013). Because mature CD62L- CD8+ T cells proliferate faster than the immature CD62L+CD8+ T cells (Buchholz et al., 2013; Kretschmer et al., 2020), it is intuitive that a larger size of a clone can be attributed to the larger proportion of the mature CD8+ T cells in the clone (Figure 1c). In contrast, NK cells proliferate slower when they differentiate to the mature CD27- phenotype. Thus, it is unclear how the similar relationship between the clone size and the composition of the clones can be understood. To probe this question further, we used a stochastic model (Figure 1a) that describes the proliferation and differentiation processes in single Ly49H+NK cells in response to MCMV infection. We do not model the MCMV infection explicitly and assume that the parameters of the model capture the effects of antigen, co-receptor, and cytokine stimulation and signaling and epigenetic modifications on the rates of differentiation and the proliferation of the cell types. In this model, immature CD27+ NK cells differentiate with a rate *r* to mature CD27- NK cells, and each cell type proliferates with rates, *k_I_* and *k_M_,* respectively. The proliferation and differentiation processes are modeled as stochastic events occurring with the above rates. We start our simulations with a single CD27+ NK cell and evaluated the number of CD27+ and CD27- NK cells at 8 days, which represent the size and the composition of a single NK cell clone. Repetitions of the stochastic simulations with the same initial configuration and rates will produce clones of different sizes and compositions due to intrinsic noise fluctuations in the stochastic simulations. We computed the correlation (*C*_size-CD27+_) between the clone sizes and the percentages of the CD27+ NK cells in each clone from our simulations. Our parameter scan of randomly sampled values of *k_I_*, *k_M_*, and *r* revealed that *C_size-CD27+_* is negative *only* when *k_I_*<*k_M_* (Figure 1d). However, the observations that CD27+ cells should grow faster than CD27- cells imply *k_I_* > *k_M_* for the NK cells. Therefore, the twostage model with no death cannot capture the heterogeneity of the NK cell clones.

**Figure 1—figure supplement 1.**
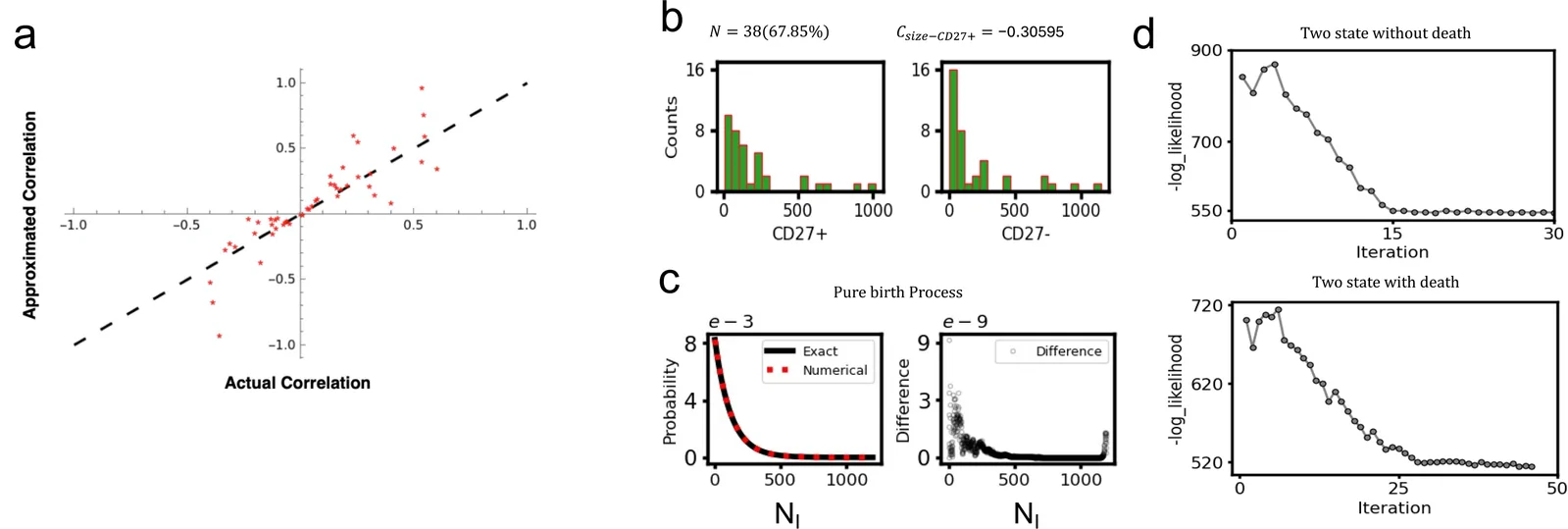
Analysis of the stochastic kinetics using the Master equation. The Master equation and its solution are described in Appendix 1. (**a**) The approximation for the correlation Csize-CD27+ maintains the sign of the actual correlation. The approximation for Csize-CD27+ (y-axis) was compared with the empirical solution (x-axis) for several parameter sets. Each parameter set is represented by a red star. The black dashed line represents the line y = x. For each parameter set, empirical Csize-CD27+ was calculated by simulating 1000 clones. (**b**) Histogram of counts of CD27+ and CD27- NK cells shown for the 38 data points used in estimating the model parameters. (**c**) (left) Comparison of the numerical solution of the Master equation at *t* = 8 days with upper bounds in NI for a pure birth process (NI → NI +1, rate bI) (dashed red) with the exact solution (solid black) with no upper bound in NI. The system starts at *t =* 0 with NI = 1; bI is taken as the largest value taken in our parameter range. (Right) shows the absolute difference in the probability distribution function of the exact solution and numerical solution. The largest difference is of the order of 10^–9^. (**d**) shows changes in the -log-likelihood with parameter iterations for the model without death (top) or with death (bottom). The minimum log-likelihood values correspond to –545.4 (top) and –514.6 (bottom). The optimal parameter values are *b_I_* = 0.97, *b_M_* = 1.07, *r* = 0.05 for two states without death, and *b_I_* = 0.97, *b_M_* = 1.7, *d_I_* = 0.15, *d_M_* = 0.92 and *r* = 0.05 for two states with death.

**Figure 1—figure supplement 2.**
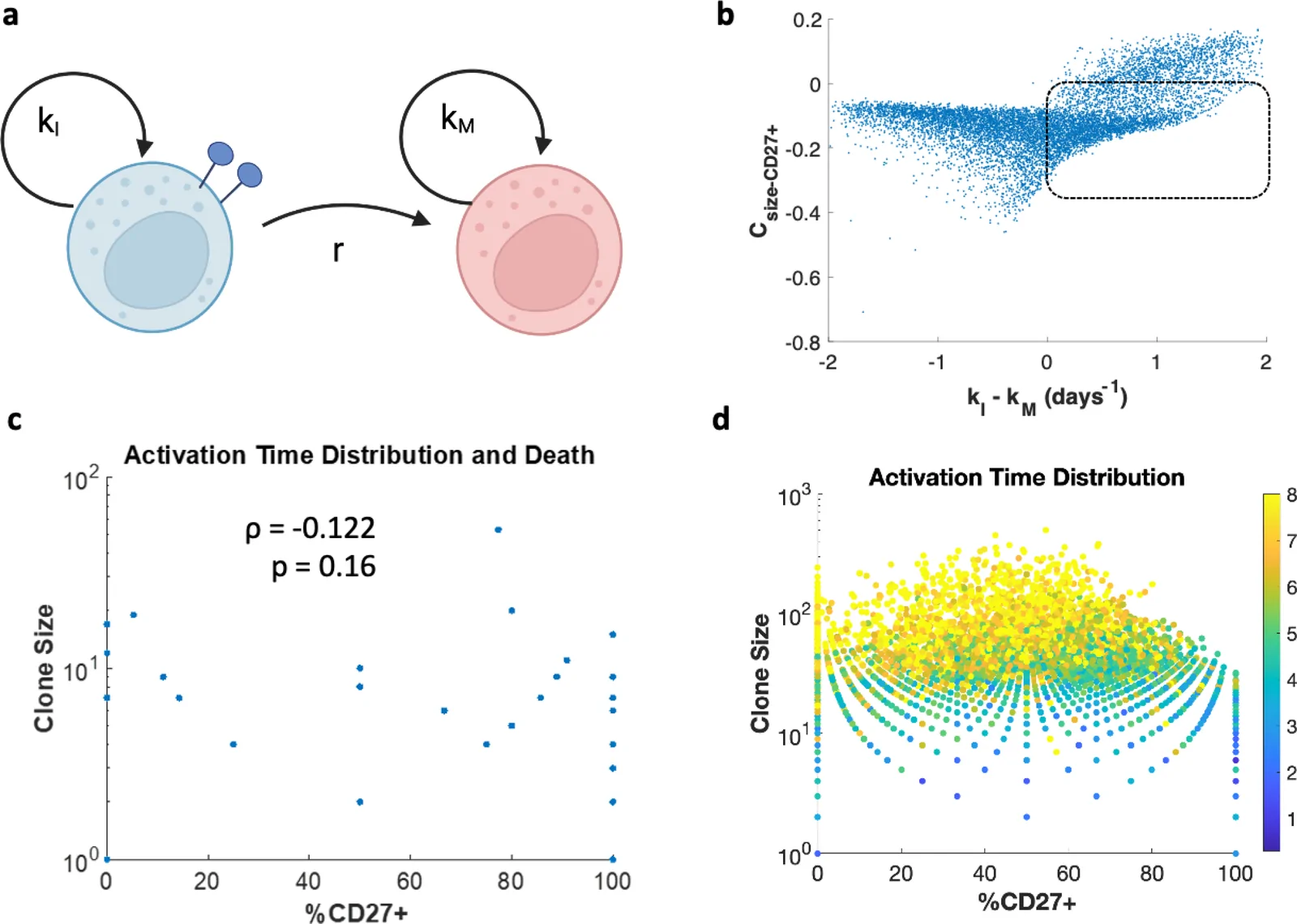
Stochasticity in the duration of NK cell activation helps contribute to negative correlations. (**a**) The model in (**a**) differs from the original two-stage model in that new CD27+ cells are introduced at varying times during the course of infection. A in the model clone is stimulated for a random length of time between 0–8 days drawn from a lognormal distribution. (**b**) Scanning different parameter values in the model shows a range of parameters (shown within the box) can produce negative values in *Csize-CD27_size-CD27+_* for rates *k_I_* > *k_M_*. The correlation was calculated for 10^4^ clones and could recreate negative correlations as well. (**c**) shows the variation of the clone sizes with the percentage of CD27+ cells in each clone for the rates taken from Appendix 3—table 1 but with growth rates in place of separate birth and death processes, where *k_I_* = 0.533, *k_M_* = 0.477, and *r*=0.135. The correlation *Csize-CD27_size-CD27+_* was calculated for 56 clones (same number of clones as the experimental data), which shows a statistically insignificant (p=0.16) negative value. (**d**) shows additional clones for the same parameter set as in (**c**) at different times to demonstrate how stochasticity in total activation time contributes to negative correlations. Color represents the length of activation, so clones that underwent division and differentiation for only one day are shown in blue.

**Figure 1—figure supplement 3.**
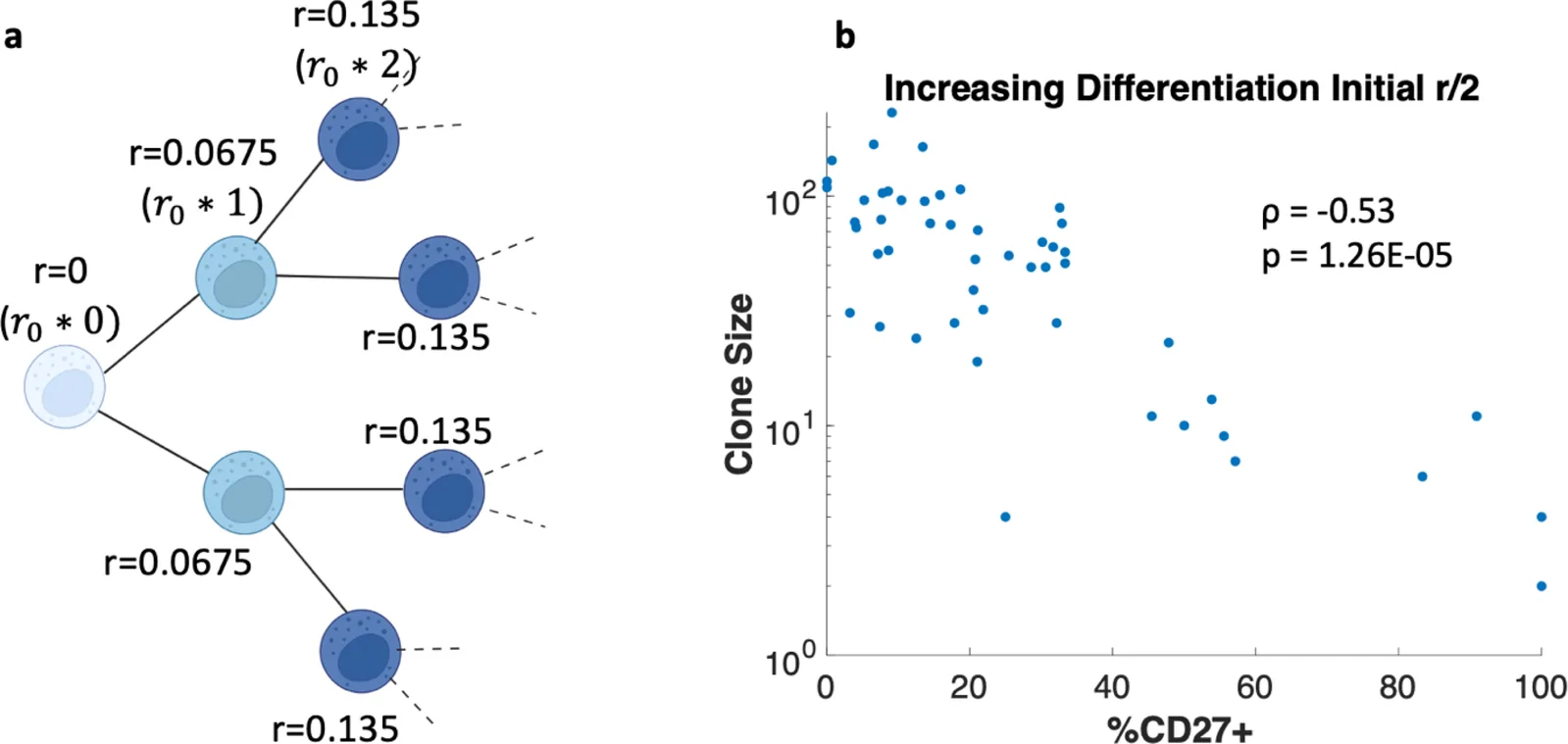
Linearly increasing differentiation rate with number of divisions results in negative correlations for a two-stage model. (**a**) A schematic representation of our model. The clonal population is initiated by a single founder cell. The daughter cells differ from the mother cell but inherit some attributes. In our case, at each cell division the rate of differentiation in the daughter cells increases linearly from that of the mother cell. In this representation, the rate of differentiation after the first division, *r*_0_=0.0675 and the rate of differentiation in the daughter cells after *n*_divisions_ divisions is given by, *r*_cell_=r_0_×*n*_divisions_. (**b**) Variation of the clone sizes with the percentage of CD27+ cells in the clones for 56 clones generated in our simulation of the model is shown (**a**). Parameters are taken from Appendix 3—table 1 but with growth rates in place of separate birth and death processes, where *k*_*I*_ = 0.533*, k*_*M*_ = 0.477, and *r*_0_*=*0.0675. The growth rate of CD27+ cells was higher than that of CD27- cells, yet the correlation is –0.532.

**Figure 1—figure supplement 4.**
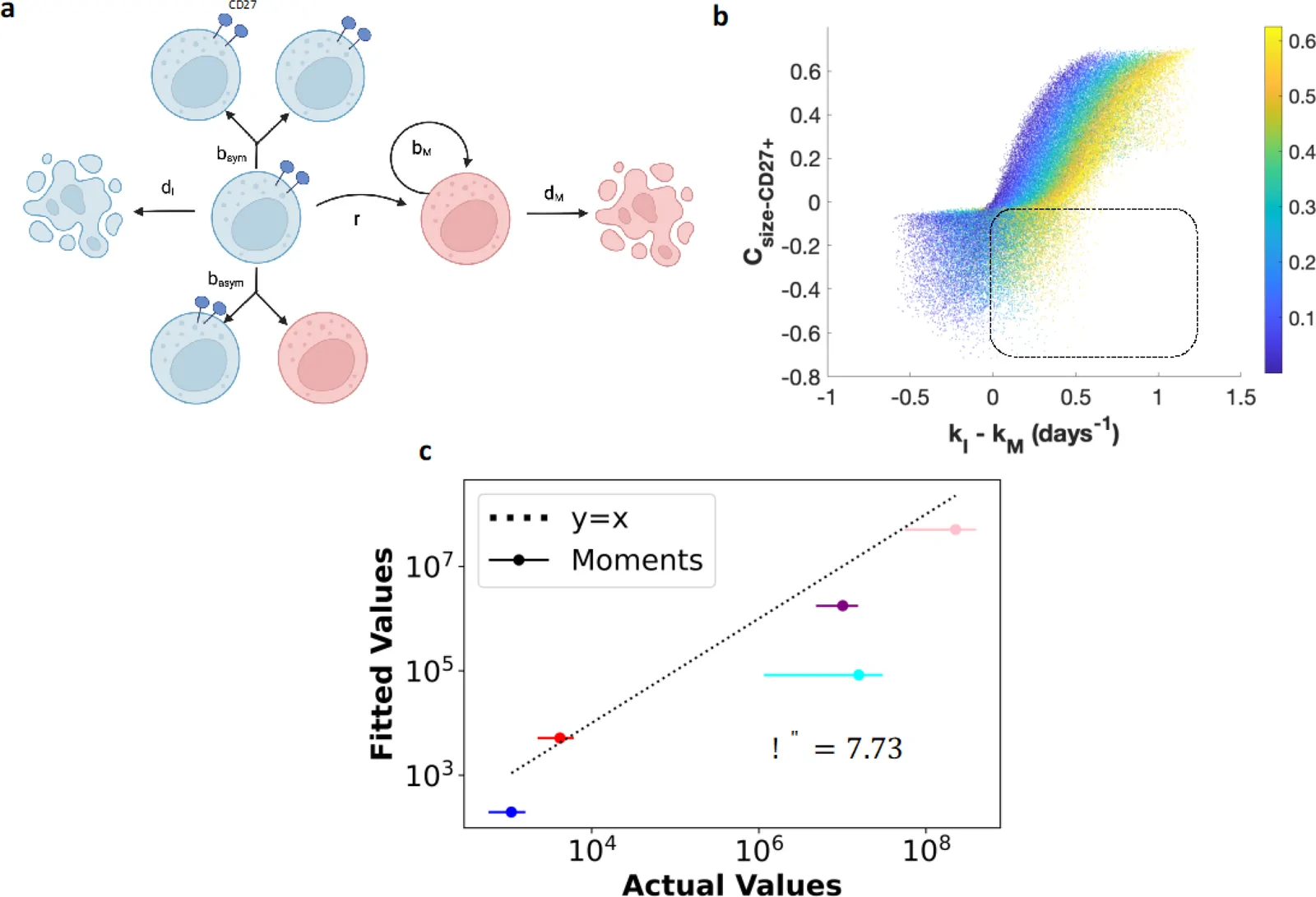
Modeling asymmetric division of CD27+ cells in a two-stage model. (**a**) Schematic representation of the two-stage model with asymmetric division. It is altered from the model in Figure 1e. So that it has two different terms for division of immature cells. Immature (CD27+) cells can divide symmetrically as before according to rate *b_sym_*, and additionally can divide asymmetrically into a single immature and single mature (CD27-) cell according to rate *b_asym_*. In this model, the growth rates are defined as *k_I_ = b_sym_ + b_asym_– d_I_* and *k_M_ = b_M_* – *d_M_*. (**b**) A parameter scan for this model where *d_I_ = d_M_ =* 0 shows that adding asymmetric division can also account for negative correlations. Each spot represents a unique parameter configuration, with color indicating the value of *b_asym_*. (**c**) Best fit of this model to the moments with constraints Δ_*k*_ > 0 and C_size-CD27+_ < –0.2.

Next, we investigated if inclusion of death of NK cells can make the model consistent with the negative correlation between the clone size and the percentage of CD27+ NK cells in each clone. We updated our proposed model to include both proliferation and death of each cell population (Figure 1e and Appendix 3—table 1). In such a model, each cell has a probability of proliferating according to birth rate *b* and probability of dying indicated by the death rate *d*. Cells can go through many proliferation events before undergoing death, which would indicate a positive net growth rate for the clone. The net growth of the CD27+ and CD27- NK cell populations are determined by *k_I_ = b_I_* – *d_I_* and *k_M_ = b_M_* – *d_M_*, respectively, where *b_I_* and *d_I_* (or *b_M_* and *d_M_*) are the proliferation and death rates of the immature CD27+ (or mature CD27-) NK cells. Repeating a parameter scan by randomly sampling the five parameters (*b_I_, d_I_, b_M_, d_M_,* and *r*) from uniform distributions and evaluating the correlation *C_size-CD27+_* shows that some parameter configurations can generate the negative values at the parameter values, *k_I_* > *k_M_* (Figure 1f). Further inspection shows that parameter values in the regime *k_I_* > *k_M_* that produce the negative correlation share a common trait: mature CD27- cells proliferate *and* die faster than immature CD27+ cells. To gain further insight into this behavior, we investigated the dependence of *C_size-CD27+_* on the parameter values and stochastic fluctuations analytically (details in Appendix 1 and Figure 1—figure supplement 1) and found that larger proliferation and death rates of the CD27- NK cells produce large stochastic fluctuations in the sizes of the CD27- cell populations. Thus, mature CD27- NK cells in a clone could undergo many proliferation events before encountering death, creating larger clones with a higher proportion of mature CD27- NK cells. Conversely, the CD27- cells could go through many death events before a proliferation event, causing the clone size to shrink (details in Appendix 1).

We investigated whether this model could accurately fit the first (means) and second (variances and covariance) moments of the measured CD27+ and CD27- NK cell populations at 8 days post-infection. We fit the model by minimizing the sum of square errors (SSE) subject to the constraints *C_size-CD27+_* < –0.2 and *k_I_ > k_M_*. This parameter configuration cannot accurately capture the moments of the clonal burst data (Figure 1g). Thus, although the model that incorporates both NK cell proliferation and death is able to produce the negative correlation between the clone size and the percentage of CD27+ NK cells in each clone, it poorly describes the distributions of the immature and mature NK cells in the clones.

To further investigate the role of NK cell death in explaining the clone size distribution, we compared the above two-stage models without or with NK cell death based on the theory of model selection using modified Akaike Information Criterion (AIC_c_) (Burnham et al., 2011). We computed the probability distribution function of the number of immature CD27+ (N_I_) and mature CD27- (N_M_) cells at any time obtained from our stochastic model by solving the corresponding Master equation numerically and computed the Likelihood function for the available clone size distribution data (details in Appendix 1), and then evaluated modified-AIC (AIC_c_) for the two models. To keep the computation in a feasible range, we used a part (38 out of 56 clone sizes) of the available data where the maximum clone size is ~1000. The subset of clone size data used here also shows a negative value of *C_size-CD27+_*. The AICc value for the two-stage model with death is over 50 points lower than that for the two-stage model without death, which clearly shows that the data favor the model with NK cell death. Although this approach only models a subset of the data, it also finds that birth and death rates for mature cells are greater than those of immature cells, and that the net growth rate of the clones of immature cells is greater than that of mature cells. Because of the computationally intensive nature of this approach and its agreement with the simpler method of fitting moments with a constraint, we continue with the later approach for subsequent models. In addition, we investigated the roles of features considered in Cyton models for T cells (Pandit and De Boer, 2019) such as heterogeneity in the duration of antigen stimulation (Figure 1—figure supplement 2), and the dependence of cell differentiation rates on the number of cell divisions (Figure 1—figure supplement 3). The model where the durations are distributed in a log-normal distribution, as well as the model where the rate of differentiation increased linearly with the number of cell divisions, both generated negative values of *C_size-CD27+_* when $k_{I}{> }k_{M}$, without cell death (Figure 1—figure supplements 2b-d and 3b). Because the distribution of the duration of activation of NK cells in CMV infections as well as the increasing rate of differentiation with increasing number of cell divisions are not established for NK cells, we did not move forward with fitting these models to clonal burst moments. We also investigated an NK cell proliferation model where the immature NK cell divides asymmetrically to generate a mature and an immature NK cell (Figure 1—figure supplement 4 and Appendix 3—table 2). T cells can undergo asymmetric division where the daughter cells proximal and distal to the antigen-presenting cell are predisposed toward the effector and the memory fates, respectively (Chang et al., 2007); though asymmetric division has not been observed in NK cells. This model generated negative values of *C_size-CD27+_* but did not provide good agreement with the moments. The above alternative models contain more parameters as well as additional mechanisms that were not validated in experiments better than the minimal model we considered here. However, these explorations suggest that additional features needed to be included in the minimal model to capture the heterogeneities observed in clonally expanded Ly49H+NK cells. The kinetics of clonal populations arising from these processes can be described by ordinary differential equations which do not permit aggregation of division and death rates; consequently, parameter estimation requires independent measurements of proliferation (e.g., Ki67) or death. In the next sections, we study some extensions of our two-stage model that can be rationalized against available experiments.

### A progressive three-stage differentiation model can quantitatively recreate clonal burst dynamics

**Figure 2.**
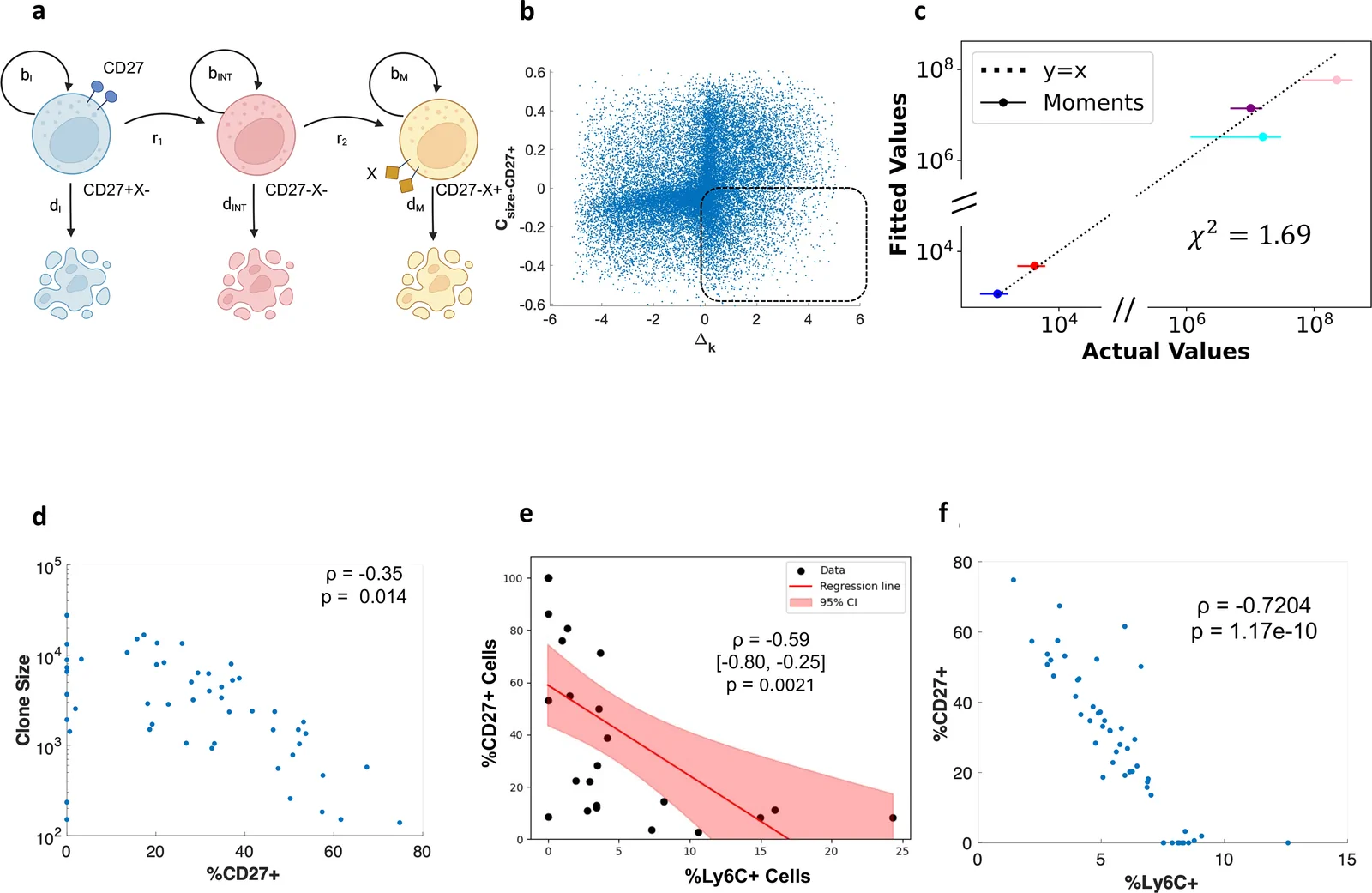
A three-stage linear model can capture experimental observations of NK cell clones if intermediate double-positive NK cells are the fastest growing subset. (**a**) Schematic representation of the three-stage model. In this model there are three stages of NK cell maturation: an immature (CD27+X-), intermediate (CD27-X-), and terminally mature (CD27-X+) subset, where the terminally mature state is marked by an expression of a yet to be determined marker protein X. Each subset has a distinct proliferation and death rate, and cells progress from immature to intermediate maturity according to rate *r_1_* and from intermediate to terminal maturity according to rate *r_2_*. (**b**) A parameter scan for the model in (**a**) shows that adding a third stage of maturation can account for the negative values in *C_size-CD27+_*. Each point represents a unique parameter configuration. Positive values of *Δ_k_* indicate that immature CD27+ cells grow faster than mature CD27- cells and vice versa. See ‘Materials and methods’ for further details. (**c**) Best fit of this model to the moments with the constraints *Δ_k_*k0 and *C_size-CD27+_* < –0.2. Circles represent the fitted values of the moments to the model. Blue and red circles represent mean populations of CD27+, and CD27- NK cells, respectively. The variances of the CD27+ and CD27- NK cells are shown in cyan and pink circles, and the covariance between the CD27+ and CD27- NK cells is shown in purple. Horizontal lines around the circles show the standard deviations of bootstrapped moments. The solid line is the line *y=x*, which demonstrates where values would lie if we had a perfect fit. (**d**) Clones stochastically simulated with Gillespie’s algorithm (‘Materials and methods’) from the best fit parameters and the resulting clone sizes, CD27+ percentages, and correlation. (**e**) Observed clonal compositions of Ly6C+ and CD27+ cells from stochastically simulated clones. (**f**) Simulated clones from the three-stage model in (**a**) at the best-fit parameter values shown in (**c**) when the three stages are taken as immature CD27+Ly6C-, mature CD27-LyC- and the terminally mature CD27-Ly6C+, respectively. Each point is a simulated clone resulting from a single NK cell.

We modified our two-stage model to include an intermediate state (Figure 2a) in the model. This reflects many investigations probing NK cell maturation, which show NK cell development goes through several maturation states and some of those states can potentially represent the proposed intermediate state in the model. A well-established NK cell maturation scheme is CD27+CD11b- → CD27+CD11b+ → CD27-CD11b+ (Chiossone et al., 2009). However, the clonal burst data show a negligible proportion of CD11b- NK cells at 8 days post-infection. Another potential maturation scheme could be CD27+KLRG1- → CD27+KLRG1+ → CD27-KLRG1+ (Kamimura and Lanier, 2015). In addition, recent single cell RNA-seq experiments show the presence of CD27-Ly6C+and CD27-Ly6C- cells at 8 days post-MCMV infection (Riggan et al., 2022), thus the intermediate state could represent a CD27-Ly6C- state as well. Here we present results for the model where the intermediate state represents a mature CD27- state; the other variations of the model are shown in Figure 2—figure supplements 1–3 and Appendix 3—tables 3 and 4. To determine parameter ranges that give rise to higher growth of mature NK cells compared to their immature counterparts, we computed the average size of a clone that originates from either a single CD27+ NK cell or single CD27- NK cell by solving ordinary differential equations (ODEs) describing the kinetics of the average NK cell populations. We constructed an effective growth difference metric *Δ_k_,* which describes the difference between the average NK cell populations at day 8 that originate from a single CD27+ or CD27- NK cell at t=0. If *Δ_k_* is positive, then CD27+ cells grow faster than CD27- cells (‘Materials and methods’). We performed a parameter scan on this three-stage model and found that some parameter configurations with *Δ_k_* > 0 can capture the negative values of *C*_size-CD27+_ (Figure 2b). We then evaluated whether this three-stage model can accurately describe the distributions of the immature and mature NK cells in clones. We fitted the means, variances, and the covariance of the NK subpopulations in the clones by varying the model parameters with constraints *Δ_k_* > 0 and *C*_size-CD27+_ < –0.2 and found that the best-fit three-stage model shows an excellent fit to the data (Figure 2c, Table 1). This best-fit parameter set generates a *C*_size-CD27+_ of –0.351, well within the confidence bounds of the correlation coefficient of the data (Figure 2d). Thus, adding an intermediate CD27- stage to the model can capture all the key features of the heterogeneity in the NK cell clonal populations.

**Table 1.** Best-fit parameters to three-stage model describing NK clonal bursts given in Figure 2a. Confidence intervals are determined by bootstrapping.

Parameter	Estimate (confidence interval)
*b*_*I*_	1.064 day^–1^ (1.051–1.215)
*b*_*INT*_	1.613 day^–1^ (1.561–1.996)
*b*_*M*_	0.700 day^–1^ (0.001–0.727)
*d*_*I*_	0.054 day^–1^ (0.052–0.232)
*d*_*INT*_	0.285 day^–1^ (0.261–0.433)
*d*_*M*_	1.370 day^–1^ (1.330–2.000)
*r*_1_	0.126 day^–1^ (0.088–0.172)
*r*_2_	0.164 day^–1^ (0.151–0.330)

**Figure 2—figure supplement 1.**
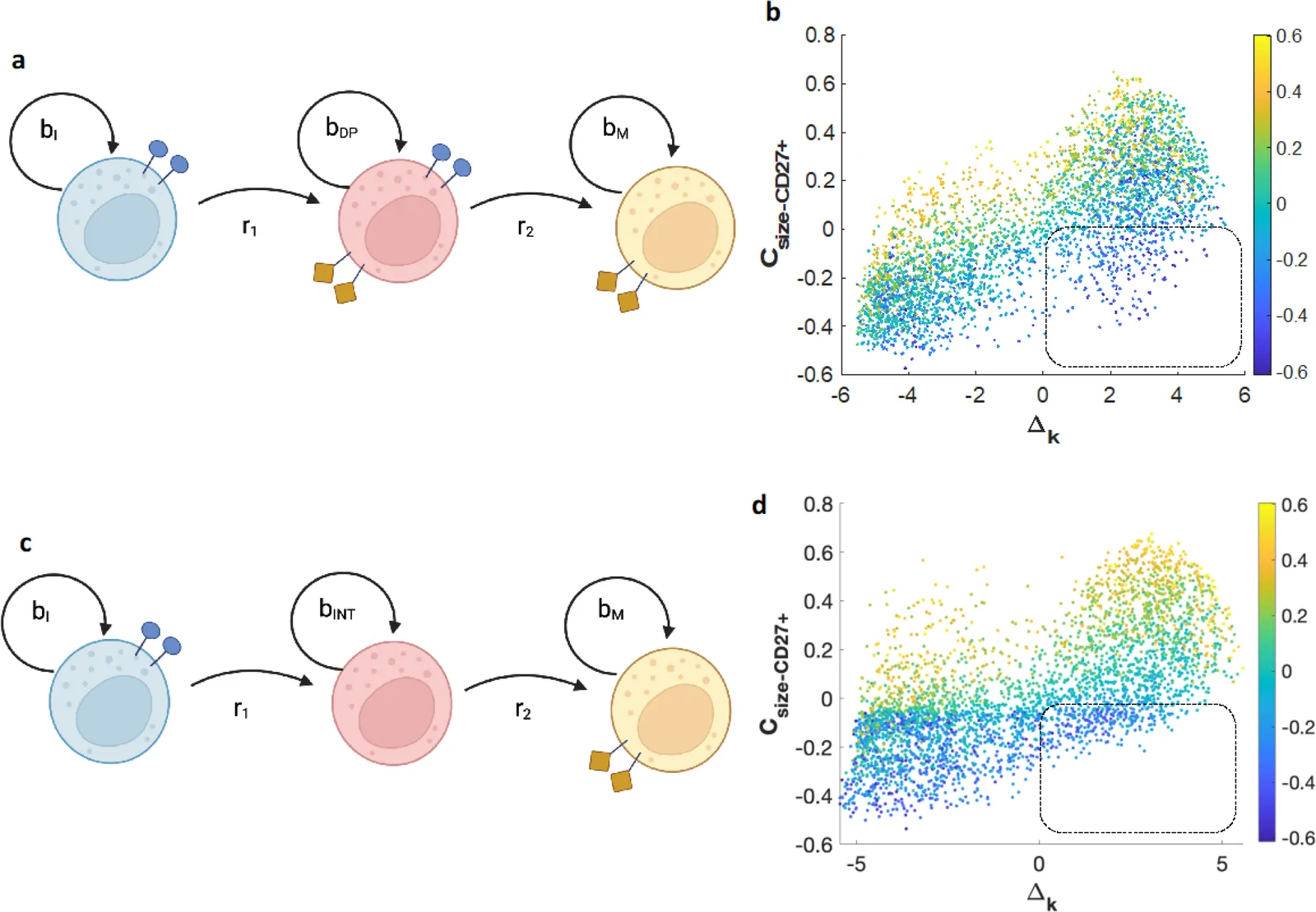
Three-stage models without death are able to capture negative correlations under the constraint Δ_k_ > 0. (**a**) Schematic representation of a three-stage model where the first two stages are CD27+ and the last stage is CD27-. (**b**) A parameter scan for the model shown in (**a**). Color represents the value *b_I_-b_DP_*We randomly initialized these simulations with an immature or double-positive cell according to the relative abundances of CD27+CD11b- and CD27+CD11b+ NK cells in the mass cytometry data. Thus, some clones are initialized with an immature cell, and others are initialized with an intermediate double-positive cell. (**c**) Schematic representation of a three-stage model where only the first stage is CD27+ and the last two stages are CD27-. (**d**) A parameter scan for the model shown in (**c**). Color represents the value *b_I_-b_INT_*.

**Figure 2—figure supplement 2.**
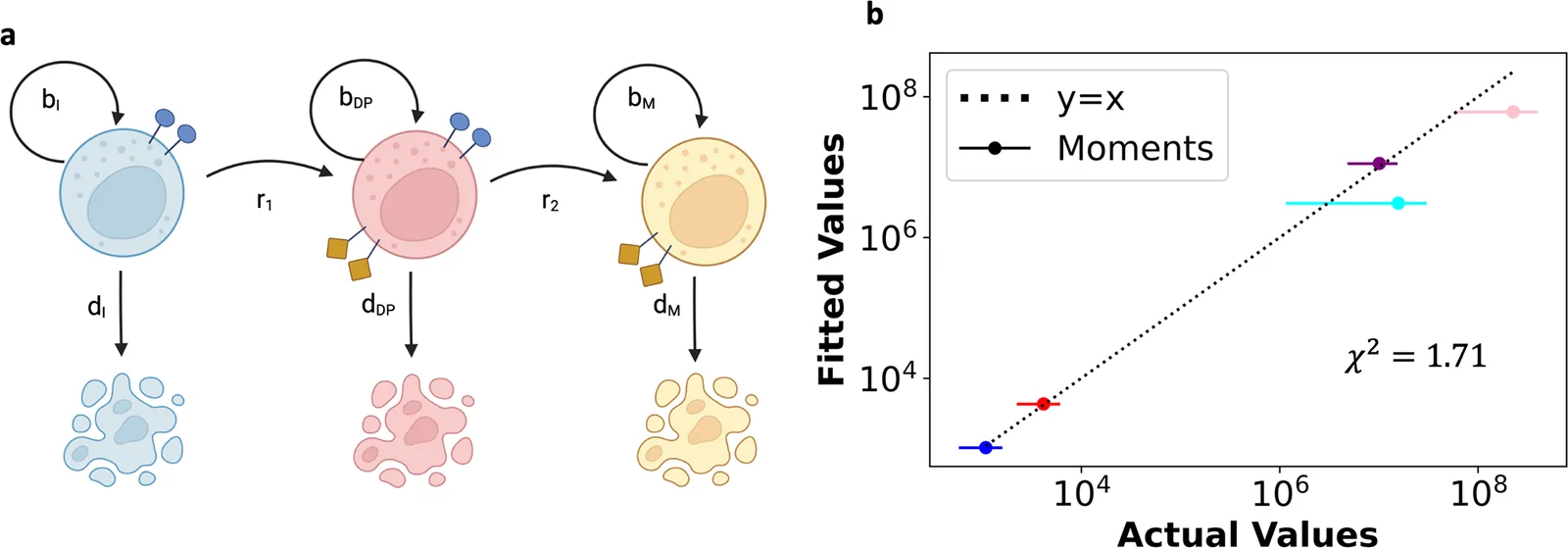
A three-stage model with two CD27+ stages quantitatively captures clonal burst moments. (**a**) Schematic representation of a three-stage model where the first two stages are CD27+ and the last stage is CD27-. Each subset undergoes both proliferation and cell death. (**b**) Best fit to the clonal burst data from the model in (**a**) given the constraints C_size-CD27+_ < –0.2 and Δ_*k*_ > 0. We used an initial condition for moment solutions and Δ_*k*_ according to the relative abundances of CD27+CD11b- and CD27+CD11b+NK cells in the mass cytometry data. Thus, some clones are initialized with an immature cell, and others are initialized with an intermediate double-positive cell.

**Figure 2—figure supplement 3.**
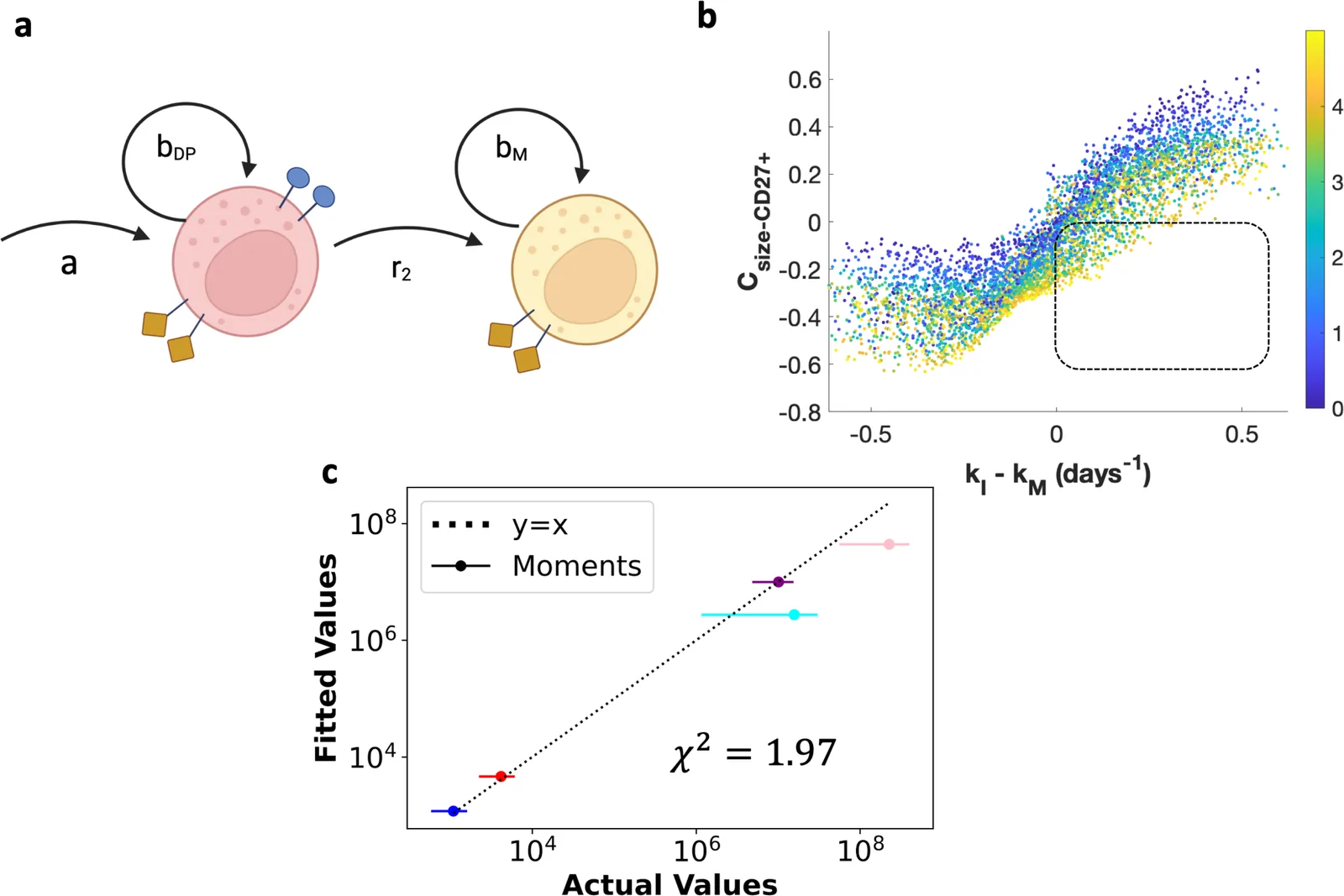
Mechanistic explanation for how a three-stage model with two CD27+ stages can create negative correlations under constraint Δ_k_ > 0. (**a**) Some parameter combinations from the parameter scan in Figure 2—figure supplement 1b have *b_I_≅r_1_*, meaning that the change in the first subset is net zero on average. Thus, we investigated a model where this population is held constant to see if negative correlations are still possible. This model is represented here, with a constant inflow *a* into a CD27+ phenotype. This model can be interpreted as having a ‘stem cell-like progenitor’. (**b**) A parameter scan for the model shown in (**a**). Color represents the value of *a*. (**c**) Best fit to the clonal burst data from the model in (**a**) given the constraints C_size-CD27+_ < –0.2 and Δ_k_ > 0.

The three-stage model above makes no assumptions about the identities of the differing phenotypes of the CD27- cell subsets. However, the best fit parameters indicate that the first CD27- subset has a high proliferation rate, and the final CD27- subset has a high death rate. Riggan et al., 2022 previously determined that, in the contraction phase, Ly6C+ NK cells differentiate from Ly6C- cells, and that Ly6C+ cells die more rapidly than Ly6C- cells. Our model shows that similar activity may be observed during the expansion phase as well, as we show higher death of Ly6C+ cells and a unidirectional maturation from Ly6C- → Ly6C+ cells. Because our analysis pertains to the expansion phase of the NK cell response to MCMV, we thus aimed to validate if the mature states proposed in the model can be identified with CD27-Ly6C- and CD27-Ly6C+ NK cells. We found that our best fit parameters correctly recreate the experimentally observed strong negative correlation between the %CD27+ and %Ly6C+ NK cells across clones (Grassmann et al., 2019), and correctly estimate that most clones have <20% Ly6C+ cells at day 8 post-infection (Figure 2e and f). This independent validation gives evidence that our parameter estimates can be meaningfully interpreted for a model where NK cells differentiate along a trajectory from CD27 +Ly6C- → CD27-Ly6C- → CD27-Ly6C+.

We sought to understand the mechanism by which the three-stage model can generate negative values for the correlation *C*_size-CD27+_ as well as quantitatively describe the heterogeneity in the NK cell populations in the clones. The two-stage model predictions for the first and the second moments for the NK cell populations are substantially less than the actual values, indicating that our best-fit parameters are not producing enough NK cells (Figure 1g). The presence of the intermediate state in the three-stage model alleviates this issue, which can rapidly grow and increase the number of CD27-Ly6C+ NK cells while the rapidly dying CD27-Ly6C- NK cells can help meet the constraint that a population of CD27- cells grows slower than one of CD27+ cells. This variation increases stochastic fluctuations in the observed numbers of CD27- cells, as some clones will have many CD27- cells from high growth of the intermediate Ly6C- subset, while other clones will have few CD27- cells from low growth of the Ly6C+ subset. Furthermore, the presence of the CD27-Ly6C+ NK cells expands the range of the model parameters that can give rise to negative values of *C*_size-CD27+_.

### Comparison of kinetics between endogenous NK cells, adoptively transferred cells, and homeostatic cells identifies potential identical maturation rates in all conditions

**Figure 3.**
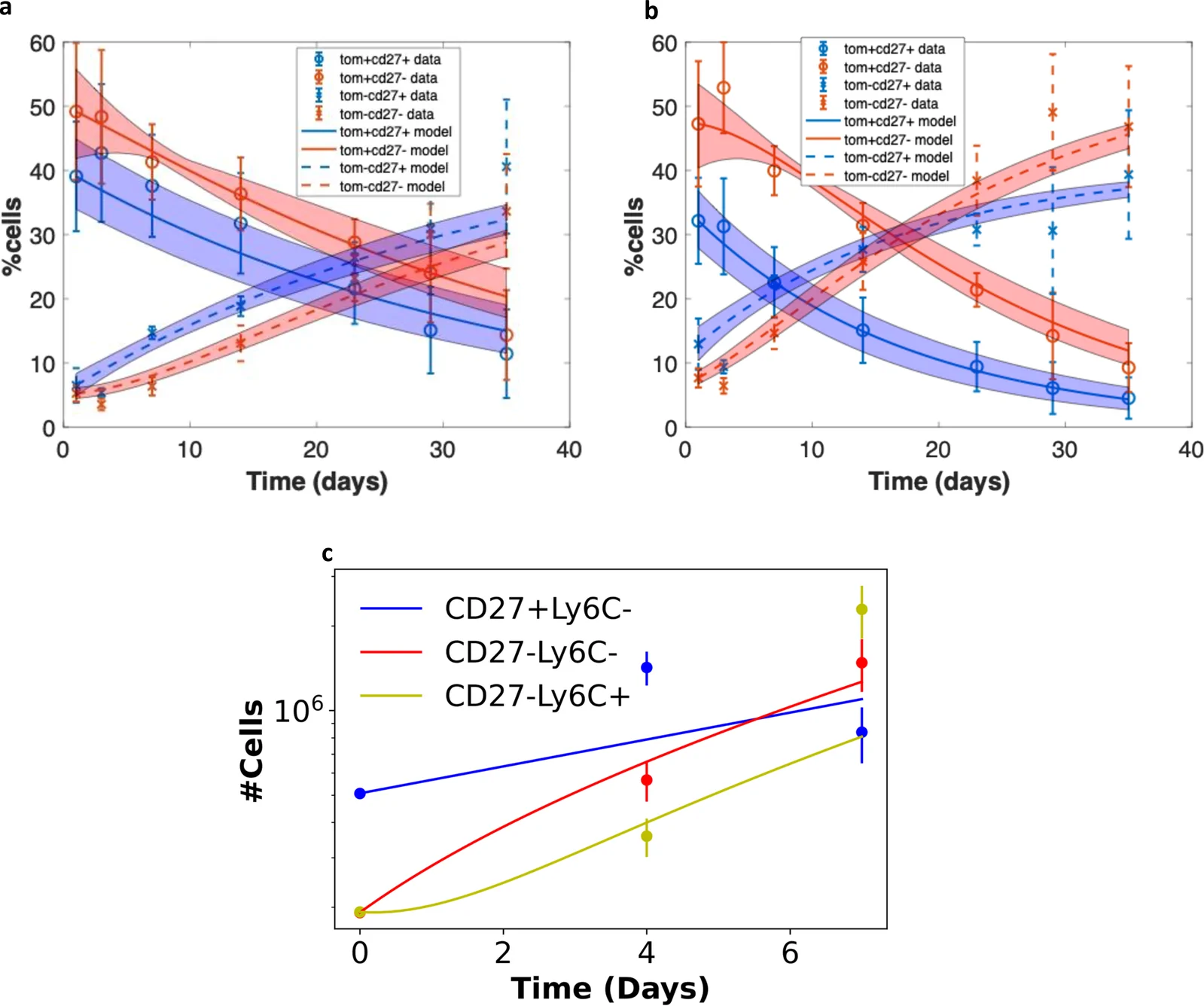
Kinetics of homeostatic NK cells and endogenous NK cells responding to MCMV infection. (**a, b**) Best fit to (**a**) Ly49H+ and (**b**) Ly49H- homeostatic NK cells. Y axis refers to percentage of the total Ly49H+ or Ly49H- population occupied by that NK subset. Circles and Xs represent the mean values of tamoxifen-induced td-Tomato positive and negative NK cells observed in the data, respectively. Solid and dashed error bars represent standard deviations around the means of tamoxifen-induced positive and negative NK abundances respectively (n = 7–9). Smooth curves represent model fits for tamoxifen-induced positive (solid) and negative (dashed) NK cells. Shaded regions refer to model 95% confidence bands, as determined by bootstrapping model fits. (**c**) Best fit to profiled endogenous NK cells responding to MCMV infection. Points represent mean cell abundances, and error bars represent standard deviations of bootstrapped means. Smooth curves represent model fit.

Here we compare the kinetics of NK cell maturation occurring in response to MCMV infection and in homeostasis. In particular, we investigated the changes in the rates of cell growth and differentiation induced by MCMV infection. First, we quantified the growth and the differentiation rates using time-stamped flow cytometry data on homeostatic NK cells in peripheral blood with a one-time treatment of tamoxifen in fluorescent NK cell reporter mice reported by Adams et al., 2021. Treating the mice with tamoxifen determines whether observed NK cells are newly populated from bone marrow or existed in peripheral blood at the onset of the infection. We fitted a two-stage CD27+ → CD27- maturation model described by ordinary differential equations (see ‘Materials and methods’) to the data collected at days 3, 7, 14, 23, 29, and 35 to estimate the growth rates of the immature CD27+ (*k_CD_*_27+_) and the mature CD27- (*k_CD_*_27_*_-_*) NK cells, the rate of differentiation (*r*) of the CD27+ to CD27- NK cells, and the rate (*λ*) with which CD27+ NK cells are populated from bone marrow. We estimated these rates both for Ly49H+ and Ly49H- NK cells. The estimated rates for Ly49H+ cells (Table 2, Figure 3a) show that a CD27+ cell population would double every 6.9 days if there were no differentiation, whereas CD27- cells have a negative growth rate with a half-life of 5.6 days. This implies that the death rate of a population of CD27- cells is greater than their proliferation rate. The differentiation rate from CD27+ → CD27- phenotypes is similar to that of the death rate of Ly49H+ CD27 cells. Approximately 1.37% of the Ly49H+ NK pool is replenished from bone marrow every day. Comparing these rates to those of the Ly49H- NK pool reveals that Ly49H- NK cells are repopulated from bone marrow at almost twice the rate (~2.4%/day). All other estimated rates for Ly49H- NK cells are within the confidence bounds of the Ly49H+ estimated rates (Table 2, Figure 3b). Given the constraint governing λ which dictates $\lambda\propto N_{CD27}$ (‘Materials and methods’), the difference in estimated λ values can be attributed to a higher percentage of CD27- cells. Next, we compared these rates with corresponding rates estimated in our three-stage model using the clonal burst data. The estimate for CD27+ → CD27- differentiation rate is equivalent between Ly49H+ NK cells in homeostasis and in MCMV infection. Our estimations for the clonal burst data (Table 1) show that varying subsets of CD27- cells have varying growth rates ranging –0.7 day^–1^ to 1.3 day^–1^ in MCMV infection, a wide range not in disagreement with the estimate for homeostatic data. Ly49H+ CD27+ cells grow ~10× faster in MCMV infection than in homeostatic conditions.

**Table 2.** Best-fit parameters to homeostatic Ly49H+ and Ly49H- NK cells. Confidence intervals are determined by bootstrapping.

Parameter	Ly49H+ estimate (confidence interval)	Ly49H- estimate (confidence interval)
λ	1.366%day (1.117–1.594)	2.412%/day (2.028–2.915)
*r*	0.125 day^–1^ (0.095–0.159)	0.147 day^–1^ (0.118–0.173)
*k*_*CD*27+_	0.097 day^–1^ (0.064–0.135)	0.088 day^–1^ (0.053–0.119)
*k*_*CD*27-_	–0.120 day^–1^ (−0.152 to –0.091)	–0.107 day^–1^ (−0.126 to –0.086)

**Figure 3—figure supplement 1.**
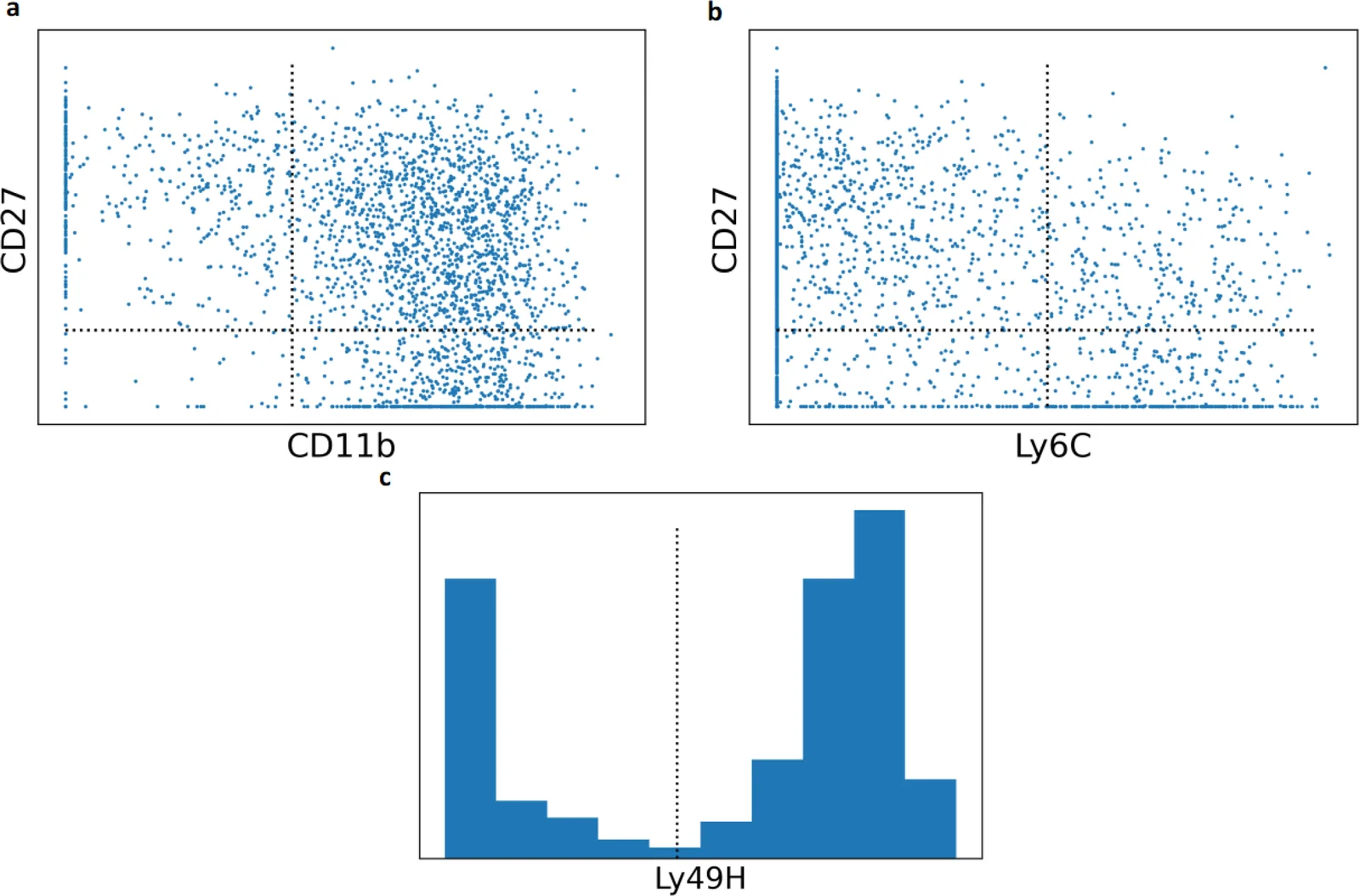
Gating strategies for mass cytometry data. All gating strategies are representative from a single mouse’s NK cells. (**a**) Gating strategy for CD27 and CD11b from Ly49H+ NK cells. (**b**) Gating strategy for CD27 and Ly6C from Ly49H+ NK cells. (**c**) Gating strategy for Ly49H.

**Figure 3—figure supplement 2.**
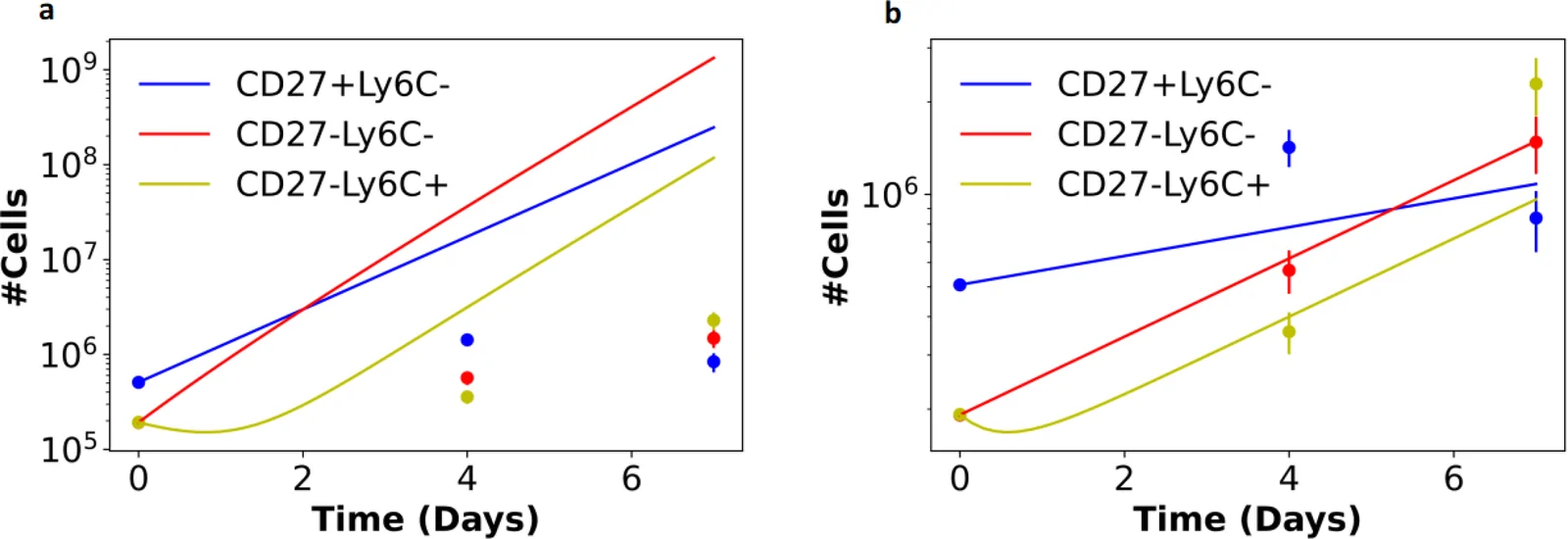
Supplemental fits to mass cytometry data. (**a**) Simulated trajectories given the best-fit parameters for the clonal burst data in Table 1. (**b**) Best fit to mass cytometry with constraints that each parameter should be less than 2 and greater than –2 (or greater than 0 for differentiation rates *r*_1_ and r_2_).

We investigated whether the kinetics of NK cell maturation in the expansion phase during MCMV infection in single-cell adoptive transfer experiments is quantitatively similar to that of an endogenous population of NK cells. To this end, we collected time-stamped mass cytometry data on C57BL/6 mice splenocytes at days 0, 4, and 7 post-MCMV infection (Potempa et al., 2022). We then gated Ly49H+ NK cells from each mouse and determined the mean relative abundances across mice of CD27+ Ly6C-, CD27-Ly6C-, and CD27-Ly6C+ subsets among the NK cell population (Figure 3—figure supplement 1). We multiplied these relative abundances by mean absolute numbers of Ly49H+ endogenous NK cells during MCMV infection from Robbins et al., 2004 to obtain time-stamped population sizes of NK cell subsets. We then modeled these data with the ODE solution in Equations S17–S25 using the parameters from Table 1 and an initial condition of the mean uninfected in vivo abundances of endogenous NK cells. The ODE result is multiple orders of magnitude above the measured abundances (Figure 3—figure supplement 2a). This indicates that the kinetics of endogenous NK cells differ significantly from that of NK cells in adoptive transfer experiments. Further indication that the kinetics differ is the expansion of cells in endogenous conditions over 7 days is less than a 10-fold increase, while clonal expansion accounts for >1000fold increase.

To further quantify the differences in the kinetic rates between the adoptively transferred and endogenous NK cells, we then fit the three-stage model with two mature stages to the endogenous cell population. We used the gated Ly49H+ populations of CD27+/Ly6C-, CD27-/Ly6C-, and CD27-/Ly6C+ NK cells and fit to a system of ODEs that describe the net growth rates of each population and the differentiation rates between them (‘Materials and methods’). The best fit to this data has unrealistic estimates for the death rate of CD27-/Ly6C+ NK cells and the differentiation rate from CD27+ → CD27- cells (Figure 3—figure supplement 2b, Appendix 3—table 5). We then attempted to constrain the fitting procedure by setting the CD27+ → CD27- differentiation rate to those of the previous estimations for the clonal burst kinetics and homeostatic conditions (Tables 1 and 2). Under this constraint, the only rate estimated to be within the confidence interval for the equivalent rate in adoptive transfer experiments is the differentiation rate from Ly6C- → Ly6C+ (Table 3, Figure 3c). The growth rate of CD27+Ly6C- cells is ~4 × faster for adoptively transferred cells, the growth rate of CD27-Ly6C- cells is 2–3× faster, and the death rate of mature cells is ~1.5× faster. However, the resulting best fit to the endogenous NK cell population does show that the intermediate CD27-/Ly6C- cell population has the fastest growth rate, and the mature CD27-/Ly6C+ cells have a large death rate, confirming the qualitative results from the adoptive transfer experiments. Thus, although the growth kinetics are different between endogenous and adoptive transfer experiments, both systems have the intermediate population proliferating rapidly and the terminally mature population dying rapidly.

**Table 3.** Best fit parameters to endogenous NK cells responding to MCMV infection with constrained *r_1_*. Confidence intervals are determined by bootstrapping.

Parameter	Estimate (confidence interval)
*k*_*I*_	0.236 day^–1^ (0.153–0.350)
*k*_*INT*_	0.537 day^–1^ (0.065–0.874)
*k*_*M*_	–0.484 day^–1^ (−1.194–0.371)
*r*_1_	0.126 day^–1^ (fixed)
*r*_2_	0.447 day^–1^ (0.000–0.874)

**Figure 4.**
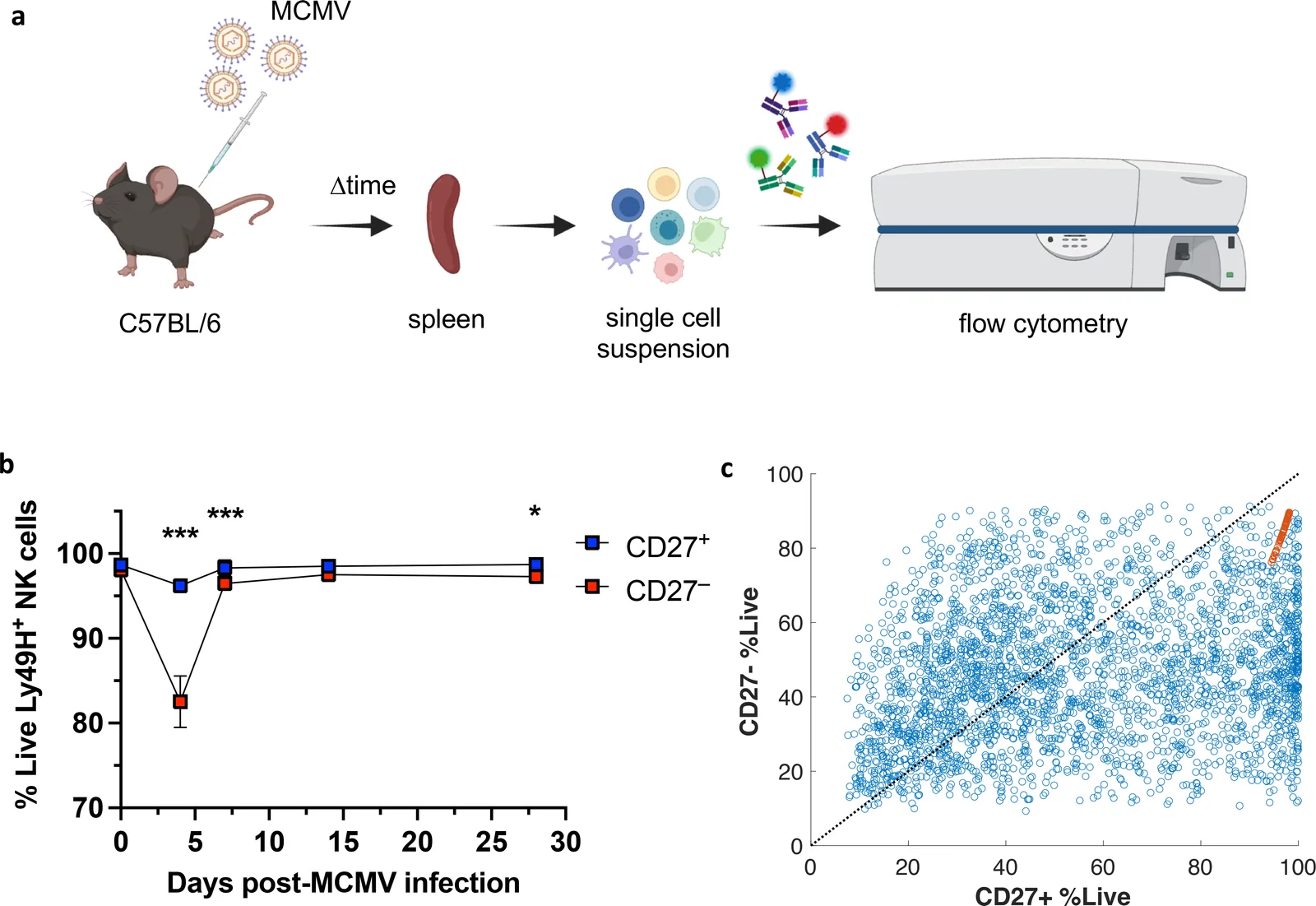
Mature CD27- NK cells undergo rapid death during the expansion phase. (**a**) Schematic of experimental setup. (**b**) Relative abundances of dead cells for CD27+ (blue) and CD27- (red) ex vivo NK cell subsets as measured by flow cytometry experiment. Data are representative of experiments with n=4–5 mice per timepoint. Graph shows means ± SEM; *p<0.033, ***p<0.001 represent statistically significant difference between CD27^+^ and CD27^–^ NK cells as determined by two-way ANOVA. (**c**) Parameter scans for determining percentage of live cells for CD27+and CD27- subsets using the model shown in Figure 4—figure supplement 1c. Blue points represent a parameter scan where birth and death rates were varied by random sampling such that the net growth rates *k*_*I*_ = *b*_*I*_ - *d*_*I*_*, k*_*INT*_ = *b*_*INT*_ - *d*_*INT*_*,* and *k*_*M*_ = *b*_*M*_ - *d*_*M*_ are equivalent to the kinetic estimates for endogenous NK cells shown in Table 3. Red points represent randomly sampled dead cell clearance rates with other rates set to those describing adoptive transfer kinetics in Table 1. Black dotted line is *y=x*, and points below this line show a higher percentage of live cells for immature CD27+ cells than for mature CD27- cells.

Both the adoptively transferred cells and endogenous population are estimated to have a large death rate for CD27- cells. To test this result, we analyzed NK cell viability in mice that were infected with MCMV at different times post-infection (Figure 4a). Surprisingly, mature (CD27-) cells indeed had a greater fraction of dead cells than immature (CD27+) cells during expansion (days 4 and 7 post-infection) (Figure 4b). To determine if the higher rates of cell death in the CD27- NK cell population in the three-stage model give rise to a higher fraction of dead NK cells, we extended our three-stage model to evaluate the number of dead mature and immature NK cells (Figure 4—figure supplement 1c), which showed that the best fit parameters for the adoptive transfer experiments are consistent with the results reported above (Figure 4c). Because we only have best-fit growth rates of the endogenous NK cells and not separate proliferation and death rates, we tested a variety of birth and death rates consistent with the best fit parameters such that *b-d=k* for each cell subset. Many of the possible configurations agree with the higher percentage of live CD27+ cells. This gives additional confirmation that CD27- cells may in fact be dying at faster rates than CD27+ cells.

**Figure 4—figure supplement 1.**
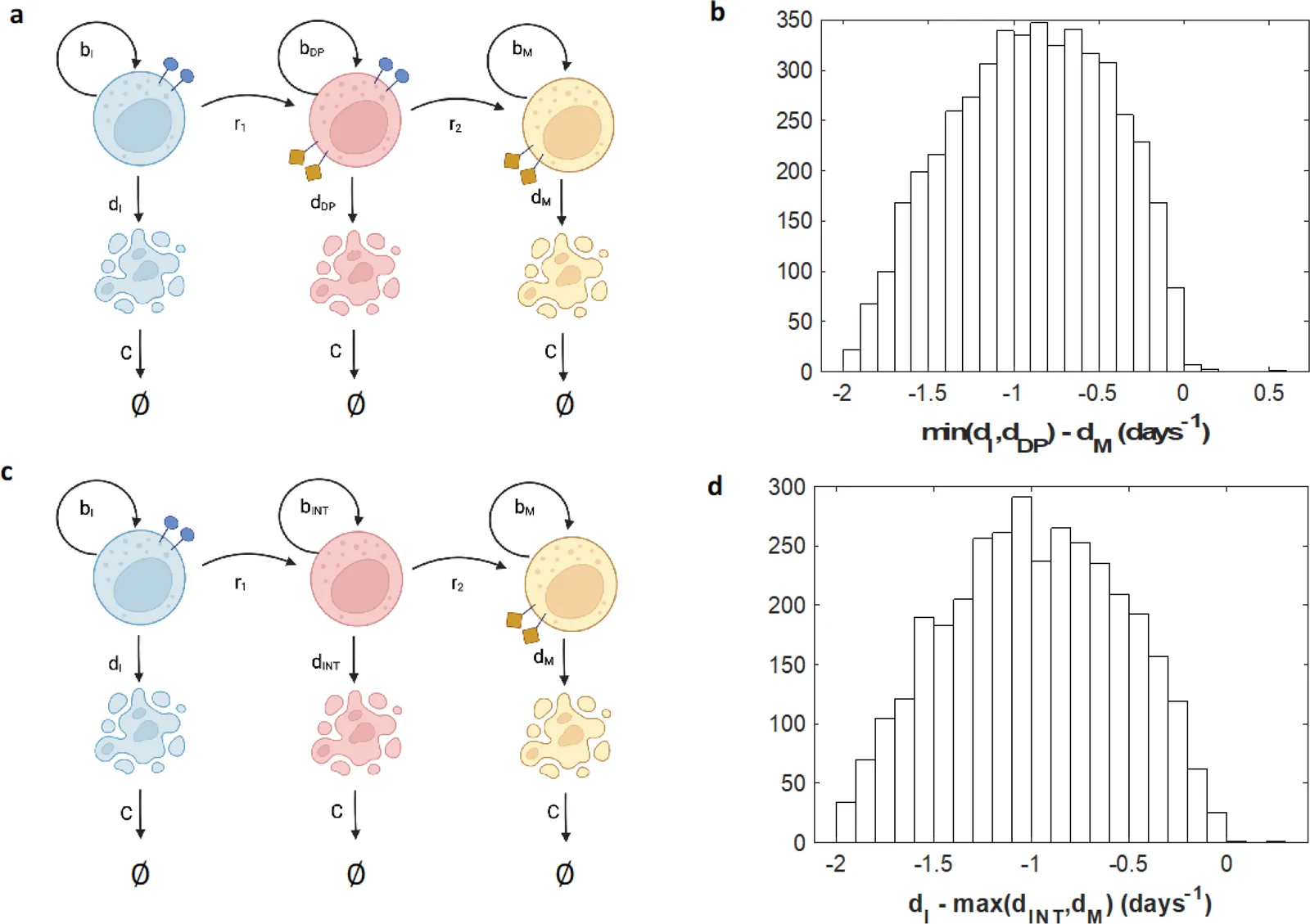
Models to measure percentage of live cells for each subset demonstrate that percentage of live cells is a good proxy for death rates. (**a**) Schematic representation of a three-stage model with two CD27+ subsets, where all dead cells are cleared at a rate *C*. Dead cell abundances are measured, and %live cells can be calculated for CD27+ and CD27- cells by dividing the abundance of live cells by the sum of live and dead cells for each subset. (**b**) A parameter scan given the initial percentages of live cells observed in the experiment in Figure 3b shows that many parameter configurations generate a higher percentage of living CD27+ cells than that of CD27- cells. Of those parameter configurations, the difference in death rates is shown in this histogram. Because all CD27- death rates are higher, this indicates that a higher %live cells for CD27+ cells requires a higher death rate of CD27- cells. (**c**) Schematic representation of a three-stage model with two CD27- subsets, where all dead cells are cleared at a rate *C*. (**d**) A parameter scan given the initial percentages of live cells observed in the experiment in Figure 3b shows that many parameter configurations generate a higher percentage of living CD27+ cells than that of CD27- cells. Of those parameter configurations, the difference in death rates is shown in this histogram. Because all CD27- death rates are higher, this indicates that a higher %live cells for CD27+ cells requires a higher death rate of CD27- cells.

## Discussion

**Figure 5.**
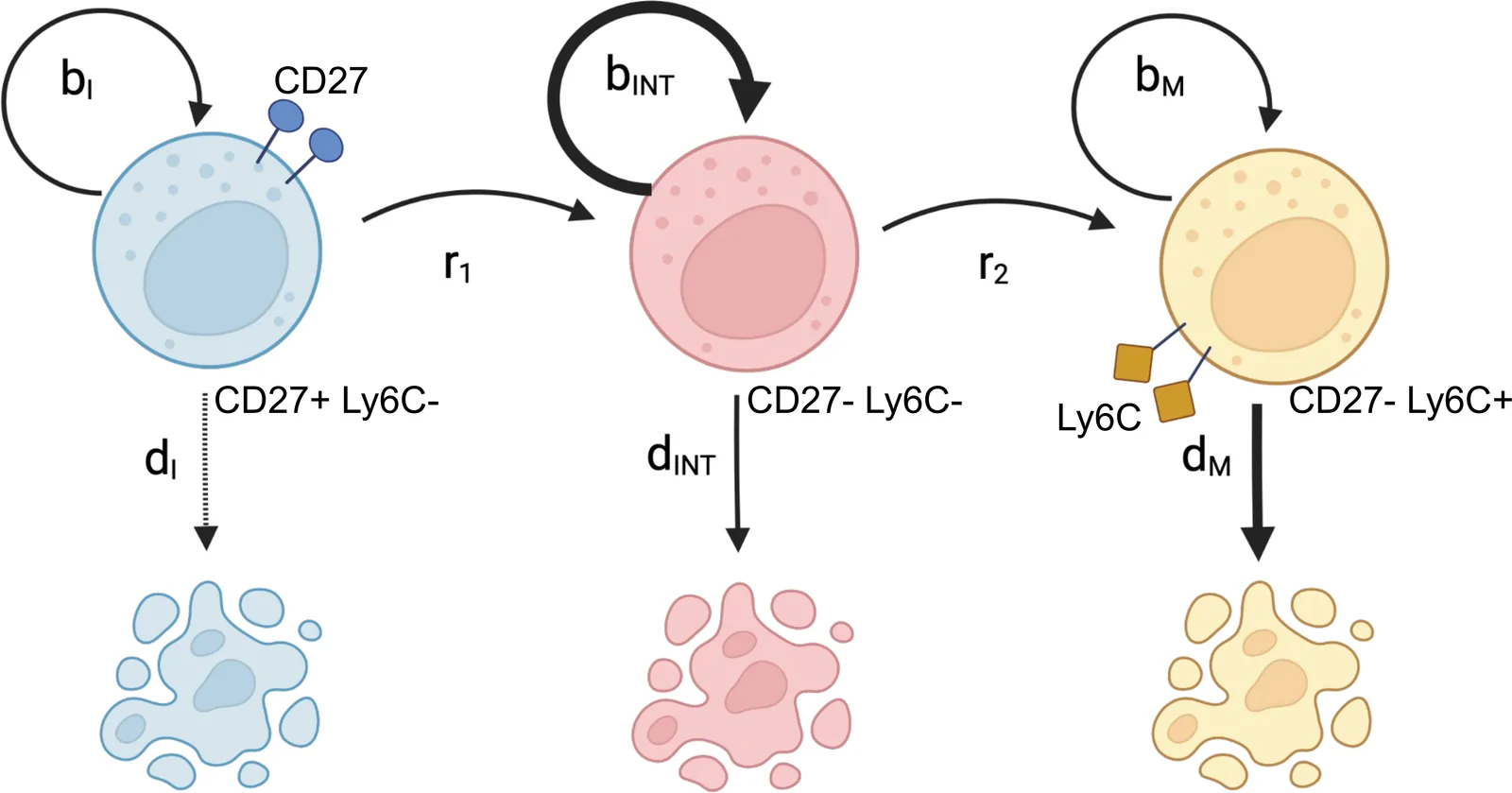
Proposed mechanism of NK clonal expansion in response to MCMV infection. Three distinct subsets of NK cells are necessary to generate full NK response to infection. The first is CD27+, Ly6C-, and moderately proliferative, with a doubling time of ~0.3 days and negligible cell death. The second is CD27-, Ly6C-, and highly proliferative, with a doubling time of ~0.2 days. The last is CD27-, Ly6C-, and prone to rapid cell death with a half-life of ~0.45 days. This picture paints CD27-Ly6C- NK cells as having a highly proliferative and highly effective phenotype, similar to effector CD8+ T cells, and CD27+Ly6C- cells as long-lived, similar to memory precursor CD8+T cells.

We employed stochastic minimal mechanistic in silico models to investigate mechanisms that underlie development of MCMV-specific NK cells at early times (0–8 days post-infection). We found Ly49H+ NK cells undergoing antigen-specific expansion follow a maturation scheme from (immature) CD27+Ly6C- → (mature I) CD27-Ly6C- → (mature II) CD27-Ly6C+, characterized by a highly proliferative mature CD27-Ly6C- phenotype and a more mature CD27-Ly6C+phenotype with higher rates of cell death (Figure 5). The differences in the rates of proliferation and cell death are crucial to give rise to the heterogeneous clones that originate from single immature CD27+ NK cells, in particular, the presence of larger sized NK cell clones composed of mature CD27- NK cells at 8 days post-infection. Similar heterogeneity in the clones of CD8+ T cells generated in the expansion phase (<8 days post-infection) from an acute antigen-specific expansion of single immature CD62L+ cells in C57BL/6 mice has been observed (Buchholz et al., 2013); however, the larger CD8+ T cell clones composed of effector CD62L- arise from the higher rate of proliferation of the CD62L- CD8+ T cells. This is in contrast with the NK cell maturation program where the larger sized clones of mature NK cell populations arise due to the interplay of uneven increase in the proliferation rates and increase in the rates of cell death as the NK cells mature, and intrinsic stochastic fluctuations occurring in these processes. Innate heterogeneity in adoptively transferred single NK cell phenotypes may also contribute to observed stochasticity. In CD8+ T cells, precursor CD62L+ cells are deemed memory precursors, matching their lowly proliferative phenotype (Kretschmer et al., 2020). An equivalent population of memory precursor NK cells has not been identified. CD27-Ly6C+ cells match previously defined phenotypes for memory NK cells and could potentially be memory precursors. However, our model shows that these cells experience rapid cell death. Therefore, for these cells to live longer to become memory cells, additional processes such as a change in environmental conditions or an epigenetic change that promotes survival in these cells at later times during the contraction phase could become relevant. This differentiation trajectory is also in contrast with CD8+ T cells, as NK cell memory precursors would be formed downstream of effector (Ly6C-) NK cells, whereas CD8+ effectors (CD62L-) are formed downstream of CD8+ T cell memory precursors (CD62L+) in acute infection. Alternately, another mature NK subset such as Ly6C-KLRG1- that is lowly proliferative and long-lived could give rise to the adaptive NK cell phenotype. Inclusion of these processes as well as the later time kinetics could be a future extension of our approach. We note a similarity of our three state NK cell maturation model with maturation of T cells in persistent (13–140 days) *T. gondii* infection in genetically resistant mice reported by Robey and colleagues (Chu et al., 2016) where the infection gave rise to a continuous production of CXCR3-KLRG1+terminally differentiated effector T cells from a quiescent memory CXCR3+KLRG1 T cells via an intermediate CXCR3+KLRG1+ state. The intermediate T cell state showed higher proliferation and increased death compared to the memory and effector T cells. However, these T cells were uniformly CD62L- in contrast to the memory and effector T cells that are produced in response to acute antigenic SIINFEKL infection.

Integration of signals in NK cells during MCMV infection leads to epigenetic remodeling affecting NK cell differentiation, proliferation, and death (Riggan et al., 2022; Rückert and Romagnani, 2024; Santosa et al., 2023). In T cells, the sum of signals received during clonal expansion has been found to regulate the number of divisions and the time of death or the division destiny in individual T cells that can arise due to a global remodeling of the transcriptional and epigenetic program (Iwata et al., 2017; Rückert and Romagnani, 2024). We considered a variant of the Cyton models (De Boer and Yates, 2023), which has been employed in modeling clonal expansion in T cells, where the rate of differentiation from the immature to the mature state depends on the number of divisions in individual NK cells. This model can give rise to negative values in the correlation between the clone size and the percentage of immature CD27+ NK cells in the absence of NK cell death, suggesting that inheritance of epigenetic states regulating cell fates in individual NK cells can play an important role in clonal expansion. Furthermore, we also find the presence of different time delays in NK cells to encounter appropriate activation signals in a pure birth model with two states can give rise to negative values in *C*_size-CD27+_ implicating a role of different activation times during the clonal expansion. The exploration of division destiny and activation times in NK cells during clonal expansion could be an exciting future direction.

NK cell clones in MCMV infection go through a smaller (~1000×) fold increase in the expansion phase compared that (~40,000×) of antigen-specific expansion of CD8+ T cells. This could be because CD8+ T cells can generate autocrine cytokines such as IL-2 that help the cells to proliferate (Kalia and Sarkar, 2018), whereas NK cells rely on other cells to make the necessary cytokines for proliferation (such as IL-2 and IL-15) and survival (such as IL-21) (Fauriat et al., 2010). Furthermore, NK cells are more dependent on cytokines such as IL-15 compared to the CD8+ T cells (DeGottardi et al., 2016). Therefore, cytokine competition for IL-15 and IL-2 might also explain increased death of mature NK cells in the expansion phase. A test of this hypothesis could be investigation of MCMV-induced NK cell maturation in *Il15+/-*mice.

We quantified the population kinetics of antigen-specific Ly49H+ NK cells during the expansion phase of MCMV infection (<8 days post-infection) using both endogenous and adoptively transferred NK cells. This antigen-specific response was further compared with the kinetics of NK cell maturation in the absence of MCMV infection. Adoptively transferred Ly49H+ NK cells grow at an increased rate compared to the endogenous Ly49H+ NK cells (~3× faster for Ly6C- cells). A possible explanation of this phenomenon is the stronger competition for resources (e.g., antigens and/or cytokines) that would occur in endogenous NK cells could prevent them from growing as quickly as the less dense population of adoptively transferred NK cells. Interestingly, our estimate of the differentiation rate of the immature CD27+ to mature CD27- NK cells showed negligible effect of MCMV infection on this process. This shows that the signaling processes governing NK cell antigen-specific expansion and cytotoxicity are not impacting transcription and translation of CD27, but rather preferential expansion of certain subsets is creating altered phenotype abundances observed in MCMV infection.

Our stochastic modeling of heterogeneous NK cell clones showed that larger stochastic variations in the mature NK cell populations arising due to higher intrinsic noise fluctuations generated by the interplay between the higher proliferation and cell death processes are important for producing large clones of mature NK cells at 8 days post-infection. This is in contrast with stochastic kinetics of CD8+ T cells where the larger clones of effector CD62L- CD8+ T cells can arise due to a higher rate of proliferation of these cells even in the absence of any cell death (Buchholz et al., 2013). Our effort to use different models to quantitatively describe the heterogeneity of NK cell clones demonstrates the critical role of the correlation between the clone size and the proportion of the mature NK cells in the clones, *C*_size-CD27+_, in screening out mechanisms that are unable to give rise to the observed heterogeneity.

## Materials and methods

### Calculation of the first and second moments and *C_size-CD27+_* for the NK cell subpopulations in the clones

The first and the second moments for the number of immature CD27+ ($N_{I}^{\left (c\right)}$) and mature CD27- ($N_{M}^{\left (c\right)}$) NK cells in *n_clone_* number of clones ({c}) are defined as follows:

$$\displaystyle  \overline{N_{I}}=1/n_{clone}\sum _{\left \{c\right \}}N_{I}^{\left (c\right)}$$

$$\displaystyle  \overline{N_{M}}=1\mathrm{/}n_{clone}\sum _{\left \{c\right \}}N_{M}^{\left (c\right)}$$

$$\displaystyle  \overline{N_{I}^{\mathrm{2}}}=1\mathrm{/}n_{clone}\sum _{\left \{c\right \}}\left (N_{I}^{\left (c\right)}\right)^{2}$$

$$\displaystyle  \overline{N_{M}^{\mathrm{2}}}=1\mathrm{/}n_{clone}\sum _{\left \{c\right \}}\left (N_{M}^{\left (c\right)}\right)^{2}$$

$$\displaystyle  \overline{N_{I}N_{M}}=1\mathrm{/}n_{clone}\sum _{\left \{c\right \}}N_{M}^{\left (c\right)}N_{I}^{\left (c\right)}$$

The correlation *C_size-CD27+_* is defined as

$$\displaystyle  C_{size-CD27+}=\left (\sigma _{T}\sigma _{\frac{N_{I}}{T}}\right)^{-1}\left [1\mathrm{/}n_{clone}\sum _{\left \{c\right \}}T^{\left (c\right)}\frac{N_{I}^{\left (c\right)}}{T^{\left (c\right)}}-\left [1\mathrm{/}n_{clone}\sum _{\left \{c\right \}}T^{\left (c\right)}\right ]\left [1\mathrm{/}n_{clone}\sum _{\left \{c\right \}}\frac{N_{I}^{\left (c\right)}}{T^{\left (c\right)}}\right ]\right ]$$

where, $ T^{(c)} = N_I^{(c)} + N_M^{(c)}, \, \sigma_T^2 = {1}/{n_{{clone}}}\sum_{\{c\}}\left(T^{(c)}\right)^2 -\left({1}/{n_{{clone}}}\sum_{\{c\}}T^{(c)}\right)^2$, and, $\sigma^2_{N_I/T} ={1}/{n_{clone}}\sum_{\{c\}}\left({N_I^{(c)}}\big/{T^{(c)}}\right)^2 -\left({1}/{n_{clone}}\sum_{\{c\}}{N_I^{(c)}}\big/{T^{(c)}}\right)^2$.

### NK cell development model

We modeled the NK cell proliferation, death, and maturation as continuous time Markov processes where a proliferation or a death event in an NK cell population increases or decreases the population number, respectively, by one cell. A differentiation event changing an NK cell from immature (*I*) to mature (*M*) decreases the NK cell population in state *I* and increases the population in state *M* simultaneously by a single NK cell. The kinetics of the system are given by the Master equation describing the time evolution of the conditional probability for the system to be in a particular state containing different numbers of immature and mature NK cells at any time *t* given a specific state at *t =* 0. The ODEs describing the kinetics of first and second moments can be derived from the Master equation. The solutions are shown in Appendices 1 and 2. The first and second moments for the clone size distributions for all our models were calculated by solving the corresponding ODEs.

### Stochastic simulations

We consider the cell proliferation, differentiation, and death processes as continuous time Markov processes that are modeled as simple birth (n # of cells → n+1 # of cells), death (n# of cells → n-1 # of cells), and differentiation (N_I_ # of immature cells, N_M_ # of mature cells → N_I_ – 1 # of immature cells, N_M_ +1 # of mature cells) processes. The propensities for the above transitions are computed for a range of rates described in Table 1, tables in the supplement, and in the parameter scan section. We use the Gillespie algorithm (Gillespie, 1977) for all our stochastic simulations. For two-stage and three-stage models, we initialized the simulation with a single CD27+ NK cell. We evolved the system until 8 days and numbers of CD27+ and CD27- cells in the NK cell clones were recorded. We calculated the correlation *C*_size-CD27+_ for the clones by averaging over 10^4^ stochastic trajectories.

### Two-stage model with differentiation rate increasing with cell division number

This is a variant of a Cyton model where the rates of proliferation, differentiation, and death individual cells are inherited from mother to the daughter cells in dividing cells. In our model, we considered a two-stage model where immature CD27+ NK cells differentiate into mature CD27- NK cells, and both immature and mature NK cells replicate with rates *k*_I_ and *k*_*M*_ (*k*_*I*_ > *k*_*M*_). In the model, we tracked the number of divisions experienced by an individual immature cell where the rate of differentiation is proportional to the number of divisions (n_division_) the cell has undergone, that is, the rate of differentiation in the cell after n_division_ is *r*_diff_ = *r*_0_ × *n*_division_, where *r*_0_ is the differentiation rate in the founder cell after the first division (Figure 1—figure supplement 3). We do not consider any cell death in the model. We implement a continuous time Monte Carlo simulation following Gillespie’s approach to simulate cell proliferation and differentiation in the system. More computational details of this multi-scale method that tracks single-cell divisions can be found in Wethington et al., 2025. We adapted the framework (Wethington et al., 2025) to include (1) a propensity for division of immature cells, (2) a propensity for division of mature cells, and (3) a propensity of differentiation that is linearly related to the number of divisions a cell has undergone.

### Two-stage model with log-normally distributed activation times

For the model with log-normally distributed activation times, we assume an NK cell received stimulation at time 8*-t* days and then expanded for a duration of *t* days until day 8. We assign based on the distribution

$$\displaystyle X{\sim }Lognormal\left (1,0.5\right)$$

$$\displaystyle t={\rm min} \left (X,8\right)$$

Each clone is evaluated for a randomly sampled *t* according to this distribution.

### Fitting model to moments

The first and second moments $f(\overrightarrow{\theta})$ for the clone size distributions obtained for two- and three-stage models were computed by solving ODEs (Equations S2–S6 in Appendix 1, and the ODEs in Appendix 2). The parameter vector $\overrightarrow{\theta}$ represents the rates for proliferation, differentiation, and death in the models. To compare the predicted moments $f(\overrightarrow{\theta})$ from the model with the actual moments *y* computed from the data, we used the cost function below:

$$\displaystyle  \chi ^{2}=\sum _{i}\left (\frac{f_{i}\left (\overrightarrow{\theta}\right)-y_{i}}{\sigma _{i}}\right)^{2}\mathrm{+}g\left (\overrightarrow{\theta}\right)$$

where *σ_i_* is the standard deviation corresponding to the data used for computing *y_i_*. A penalty function, *g*($\overrightarrow{\theta}$), is introduced to implement the constraint imposed by the sign of the correlation Csize*_-CD27+_*. We set *g*($\overrightarrow{\theta}$) to a high value (=10^4^) when *C_size-CD27+_* > –0.2 at any value of Δ*_k_* or *C_size-CD27+_* < 0 and Δ*_k_*k0; otherwise, *g*($\overrightarrow{\theta}$)=0. This constraint describes the experimental observation that immature CD27- NK cells grow faster than mature CD27+ NK cells in the expansion phase. *σ_i_* is calculated by taking the standard deviation of each moment across 10,000 bootstrapped samples of the clones. Minimization of the cost function was performed with the *lmfit* python package, using the ‘least_squares’ Trust Region Reflective algorithm. Parameter search space was confined from 0.0 to 2.0 day^–1^ for all parameters. We also performed a parameter fitting without any constraint on Δ_k_, instead fitting the ratio of cells arising from a 1:1 mix of CD27+/CD27- NK cells following the experiments in Flommersfeld et al., 2021, adding one additional data point and residual to the model. The resulting best-fit parameters represent Δ_*k*_ < 0, thus we reasoned these estimated parameters do not capture the NK cell expansion observed in experiments (Appendix 3—table 6). This is in line with the previous findings that setting CD27- as the faster growing population can match observed moments data, but this must be balanced with the finding that CD27+ cells grow faster (i.e., Δ_*k*_ > 0).

### Effective growth rate for three-stage system

We developed a metric to quantify the difference in the growth rate of immature CD27- and mature CD27+ NK cells for the three-stage model. We computed mean populations of the NK cell subsets at day 8 by solving Equations S17–S25 in Appendix 2 for two different initial conditions. In one case, we initialized with the immature CD27+Ly6C- cells, and in the other case we initialized with equal proportions of mature CD27-Ly6C- and mature CD27-Ly6C + cells. We then computed the ‘effective growth difference’ (Δ*_k_*) by evaluating the difference in the mean populations of the immature CD27+ and the mature CD27- NK cells at day 8. We performed an asinh(Δ_*k*_) transformation to help visualization of the data in the graphs shown in the manuscript.

### Parameter scans

For parameter scans showing the relationship between growth rates and the correlation between clone size and %CD27+ cells, each parameter is randomly selected from a uniform distribution from 0 to 1.6 day^–1^. For each parameter set, 10^4^ Gillespie simulations were carried out to compute the correlation *C*_size-CD27+_ for the NK cell clones at day 8. We performed scans for 10^5^ different parameter sets.

### Ordinary differential equation fits to homeostatic data

The growth and differentiation kinetics of populations of CD27+Tom + (*n*_1_), CD27-Tom+ (*n*_2_), CD27+Tom- (*n*_3_), and CD27-Tom- (*n*_4_) cells in the periphery in homeostasis are given by the ODEs below:

$$\displaystyle \frac{dn_{1}}{dt}=k_{CD27+}n_{1}-rn_{1}$$

$$\displaystyle \frac{dn_{2}}{dt}=k_{CD27-}n_{2}+rn_{1}$$

$$\displaystyle \frac{dn_{3}}{dt}=k_{CD27+}n_{3}-rn_{3}+\alpha $$

$$\displaystyle \frac{dn_{4}}{dt}=k_{CD27-}n_{4}+rn_{3}$$

The CD27+ and CD27- cells grow with net rates *k*_*CD*27+_ and *k*_*CD*27-_, respectively, and the differentiation CD27+ → CD27- occurs at a rate, *r*. The net rates (k_*CD*27+_ and *k*_*CD*27-_) of growth or loss relate to clonal rather than cellular dynamics. The influx of immature CD27- cells from the bone marrow to the periphery occurs at a rate *α*. At homeostasis, it is reasonable to assume that the sum of all Ly49H+ and the sum of all Ly49H- NK cells were assumed to be constant through time, that is,

$$\displaystyle  n_{TOT}\left (t=0\right)=n_{TOT}\left (t\right)=\sum _{i}n_{i}\left (t\right)$$

The kinetics of the percentages (*N*_*i*_ =100 × (*n*_*i*_/*n*_*TOT*_)) of each cell type follow the ODEs below:

$$\displaystyle \frac{dN_{1}}{dt}=k_{CD27+}N_{1}-rN_{1}$$

$$\displaystyle \frac{dN_{2}}{dt}=k_{CD27-}N_{2}+rN_{1}$$

$$\displaystyle \frac{dN_{3}}{dt}=k_{CD27+}N_{3}-rN_{3}+\frac{\alpha }{n_{TOT}}$$

$$\displaystyle \frac{dN_{4}}{dt}=k_{CD27-}N_{4}+rN_{3}$$

We define $\lambda =\frac{\alpha }{n_{TOT}}$, which describes the influx rate of either the total Ly49H+ or total Ly49H- population as a percentage of the given population. This makes the ODEs which we fit:

$$\displaystyle \frac{dN_{1}}{dt}=k_{CD27+}N_{1}-rN_{1}$$

$$\displaystyle \frac{dN_{2}}{dt}=k_{CD27-}N_{2}+rN_{1}$$

$$\displaystyle \frac{dN_{3}}{dt}=k_{CD27+}N_{3}-rN_{3}+\lambda $$

$$\displaystyle \frac{dN_{4}}{dt}=k_{CD27-}N_{4}+rN_{3}$$

We assume the total number of NK cells and the relative abundances of varying subsets do not change with time in homeostasis; therefore,

$$\displaystyle \frac{d\left (N_{1}+N_{2}+N_{3}+N_{4}\right)}{dt}=k_{CD27+}\left (N_{1}+N_{3}\right)+k_{CD27-}\left (N_{2}+N_{4}\right)+\lambda =0$$

$$\displaystyle \frac{d\left (N_{1}+N_{3}\right)}{dt}=k_{CD27+}\left (N_{1}+N_{3}\right)-r\left (N_{1}+N_{3}\right)+\lambda =0$$

$$\displaystyle \frac{d\left (N_{2}+N_{4}\right)}{dt}=k_{CD27-}\left (N_{2}+N_{4}\right)+r\left (N_{1}+N_{3}\right)=0$$

These constraints lead to equalities between parameters,

$$\displaystyle r=-\frac{k_{CD27-}N_{CD27-}}{N_{CD27+}}$$

$$\displaystyle \lambda =-\left (k_{CD27-}N_{CD27-}+k_{CD27+}N_{CD27+}\right)$$

Thus, by estimating only *k_CD_*_27_*_+_* and *k_CD_*_27_*_-_*, and using the mean relative abundances of CD27+ and CD27- cells across mice and time, we can solve all four rate parameters in the homeostatic state.

### Ordinary differential equation fits to mass cytometry data

We gated Ly49H+ NK cells in the CyTOF studies into three subsets: CD27+Ly6C-, CD27-Ly6C-, and CD27-Ly6C+. Details of the gating are described in Figure 3—figure supplement 1. We describe the net growth and differentiation kinetics of the populations the immature CD27+Ly6C- (*N*_*I*_), mature CD27-Ly6C- (*N*_*INT*_), and mature CD27-Ly6C+ (*N*_*M*_) by the ODEs below:

$$\displaystyle \frac{dN_{I}}{dt}=k_{I}N_{I}-r_{1}N_{I}$$

$$\displaystyle d\frac{N_{INT}}{dt}=k_{INT} N_{INT} +r_{1}N_{I}-r_{2}N_{INT}$$

$$\displaystyle \frac{dN_{M}}{dt}=k_{M}N_{M}+r_{2}N_{INT}$$

where *k_I_*, *k_INT_*, and *k_M_* denote the net rates of growth (or loss) for *N_I_*, *N_INT_*, and *N_M_*, respectively. The net rates (*k_I_*, *k_INT_*, and *k_M_*) of growth or loss relate to clonal rather than cellular dynamics. The rates of differentiation for CD27+Ly6C- → CD27-Ly6C- and CD27-Ly6C- → CD27-Ly6C+ are given by *r*_1_ and *r*_2_, respectively. Since the above system of ODEs is autonomous and linear, we can obtain the time-dependent solutions analytically and exactly. The analytical solution was numerically evaluated for different values of the rate parameters represented by $\overrightarrow{\theta }$. The analytical solutions were evaluated numerically for specific rate constants and initial conditions. We used the estimated population sizes (details in the main text) for the above NK cell subsets in the CyTOF data at *t*=0 as the initial conditions and computed the population sizes at day 4 ({*N*_i_($\overrightarrow {\theta }$, *t=4d*)}) and day 7 ({*N*_i_($\overrightarrow{\theta }$, *t=7d*)}) by solving the above ODEs for a parameter set $\overrightarrow{\theta }$. We then estimated the parameters by $\overrightarrow{\theta }$ by fitting the population sizes at day 4 ({*y*_i_($\overrightarrow{\theta }$, *t*=*4d*)}) and day 7 ({*y*_i_($\overrightarrow{\theta }$, *t=7d*)}) using the cost function below

$$\displaystyle  \chi ^{2}=\sum _{i}\left (\frac{N_{i}\left (\overrightarrow{\theta },t=4d\right)-y_{i}\left (t=4d\right)}{\sigma _{i}}\right)^{2}+\left (\frac{N_{i}\left (\overrightarrow{\theta },t=7d\right)-y_{i}\left (t=7d\right)}{\sigma _{i}}\right)^{2}$$

### Bootstrapping confidence intervals for parameter estimates

To assess the confidence intervals for parameter estimates, we randomly sampled the clones with replacement with equal probability, calculated each moment and its standard deviation, and fit the moments as described above. We repeated the process 1000 times, yielding 1000 estimates for the parameters *θ*. We then sorted the parameter estimates by magnitude and took the 2.5th and 97.5th percentiles for each. These estimates made up the 95% confidence intervals reported here.

For estimations of the homeostatic and endogenous NK populations, we sampled a population of mice before sampling errors in measurements for each bootstrap estimate.

### Bootstrapping confidence intervals for model fits

The model fits in Figure 3a and b have confidence intervals around them. These are calculated by bootstrapping parameter estimates as described above, and simulating a trajectory for each bootstrapped parameter estimate set. At each time *t*, we take the 2.5th and 97.5th percentiles of the model trajectory for each cell type. These make up the lower and upper limit for the 95% confidence interval at time *t*.

### Animals and MCMV infection

C57BL/6 (B6) B6 mice were purchased from the National Cancer Institute at 6 weeks of age and housed in the specific pathogen-free animal facility at the University of California San Francisco (UCSF) in accordance with the guidelines of the Institutional Animal Care and Use Committee. Experiments used mice of both genders, aged 8–12 weeks.

B6 mice were infected with 1000 plaque-forming units (PFU) of Smith strain MCMV via intraperitoneal injection. Spleens were acquired at different time points following MCMV infection and examined by flow cytometry.

### Flow cytometry

Splenocytes from uninfected or infected mice were processed into single-cell suspensions, RBC lysed, and then stained for flow cytometry. Surface staining was performed using pre-conjugated antibodies in the presence of the viability marker ZombieRed (BioLegend) and were then analyzed on an LSR II cytometer (BD Biosciences). The antibodies used for flow cytometry were: CD3ε (145-2C11), CD19 (6D5), CD49b (DX5), NK1.1 (PK136), KLRG1 (2F1), Ly6C (HK1.4), CD27 (LG.3A10), and Ly49H (3D10). All antibodies were purchased from BioLegend, BD Biosciences, or ThermoFisher Scientific. An unconjugated anti-CD16/CD32 antibody (clone 2.4G2, UCSF Antibody Core) was used to block non-specific binding.

### Mass cytometry

Samples were prepared for mass cytometry as previously described (Potempa et al., 2022). Briefly, splenocytes from each mouse were obtained by mechanical dissociation through a 40 μm filter, treated with ACK lysis buffer, and resuspended in PBS + 5 mM EDTA (PBS/EDTA). Cells were stained for viability by mixing 1:1 with PBS/EDTA + 100 mM cisplatin (Enzo Life Sciences) for 1 minute before quenching with PBS/EDTA +0.5% BSA (Staining Media). One million splenocytes from each mouse were stained with metal-conjugated antibodies against CD16 (Nd144) and CD32 (Eu153) for 15 minutes at room temperature in Staining Media before supplementation with an Fc blocking Ab (clone 2.4G2). After washing in Staining Media, cells were stained with a master mix of 35 additional metal-conjugated antibodies (Appendix 3—table 7) for 30 minutes at room temperature. At the conclusion of the staining period, cells were washed in PBS/EDTA before fixation with PBS/EDTA +3.2% paraformaldehyde and stored at 4°C for 3–5 days. One day prior to data acquisition, samples were barcoded using a 20-plex Pd barcoding kit (Fluidigm) per manufacturer protocol, pooled, and fixed again, overnight at 4°C, in a PBS/EDTA +3.2% paraformaldehyde solution supplemented with a 1:1000 dilution of a 191/193 DNA intercalator (Fluidigm). Just prior to acquisition, barcoded samples were pelleted, washed in double-deionized water, and resuspended in double-deionized water with a 1:20 dilution of EQ four Element Calibration Beads (Fluidigm). Data were acquired using a CyTOF2-Helios mass cytometer (Fluidigm), which were then normalized and debarcoded prior to analysis.

## Figures and Tables

**Appendix 1—scheme 1.**
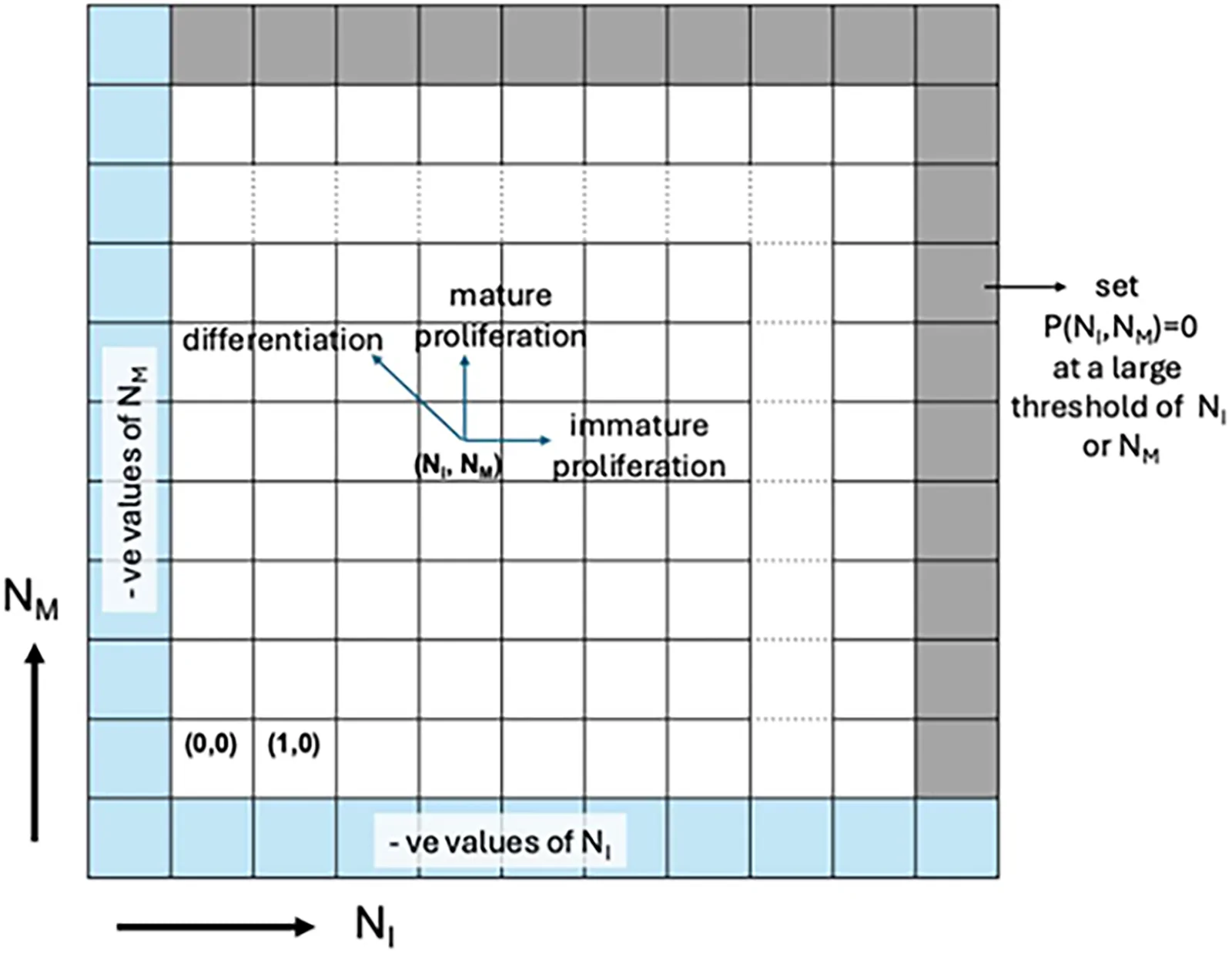
Schematic diagram showing the values of the numbers of immature (*N*_*I*_) and mature (*N*_*M*_) for which the Master equation was solved numerically. The boundary layers ($N_{I}^{ub},0\leq N_{M}\leq N_{M}^{ub}$ and $0\leq N_{I}\leq N_{I}^{ub},N_{M}^{ub}$) at the upper bounds of the numbers used to make the computation feasible are marked in grey. The values where *N*_*I*_ and *N*_*M*_ assume negative values are shown in blue.

## Data Availability

We used previously published data for this work. The data and code for the work are available at https://github.com/jayajitdas/NK-cell-clonal-dynamics (copy archived at Das, 2026). Any additional details can be requested by contacting the corresponding authors.

## Additional files

MDAR checklist

## Appendix 1

### Analysis of the stochastic kinetics of NK clonal expansion

#### Two-stage system

We consider the model shown in Figure 1a. The immature (state I) CD27+ NK cells differentiate into mature (state M) CD27- NK cells with a rate *r*, that is, $I \overset{r}{\rightarrow} M$. The immature and mature NK cells proliferate with rates $k_{I}$ and $k_{M}$, described by the reactions $I \overset{b_{I}}{\rightarrow} I\mathrm{+}I$ and $M \overset{b_{M}}{\rightarrow} M\mathrm{+}M$, respectively. We will also consider a model with cell death described by reactions $I \overset{d_{I}}{\rightarrow} \phi $ and $M \overset{d_{M}}{\rightarrow} \phi $. The stochastic kinetics of these processes are given by the Master equation describing the time evolution of the probability distribution $P_{N_{I},N_{M}}\left (t\right)$ of the numbers of immature (*N*_*I*_) and mature (*N*_*M*_) NK cells at any time *t* given the initial condition $N_{I}\left (t=0\right)=1,N_{M}\left (t=0\right)=0$. Note that the model without death may be described as the model with death with $d_{I}=d_{M}=0$.

We can easily derive the Master equation for $P_{N_{I},N_{M}}\left (t\right)$:

(S1) $$\displaystyle  \frac{dP_{N_I,N_M}}{dt} =\, & b_I(N_I-1)P_{N_I-1,N_M} +d_I(N_I+1)P(N_I+1,N_M) \\ &+r(N_I+1)P_{N_I+1,N_M-1} + b_M(N_M-1)P_{N_I,N_M-1} \\ &+d_M(N_M+1)P_{N_I,N_M+1} -\big((b_I+d_I+r)N_I \\ &+(b_M+d_M)N_M\big)P_{N_I,N_M} $$

The ODEs describing the average values (indicated by overbars) of *N*_*I*_ and *N*_*M*_ can be derived from the above Master equation in Equation S1.

(S2) $$\displaystyle \frac{d\overline{N}_{I}}{dt}=\left (b_{I}-d_{I}-r\right)\bar{N}_{I}$$

(S3) $$\displaystyle \frac{d\overline{N}_{M}}{dt}=r\overline{N}_{I}+\left (b_{M}-d_{M}\right)\overline{N}_{M}$$

Second order:

(S4) $$\displaystyle \frac{d\overline{N_{I}^{{2}}}}{dt}=2\left (b_{I}-d_{I}-r\right)\overline{N_{I}^{{2}}}+\left (b_{I}+d_{I}+r\right)\overline{N}_{I}$$

(S5) $$\displaystyle \frac{d\overline{N_{M}^{{2}}}}{dt}=2\left (b_{M}-d_{M}\right)\overline{N_{M}^{{2}}}+\left (b_{M}+d_{M}\right)\overline{N}_{M}+2r\overline{N_{I}N_{M}}+r\overline{N}_{I}$$

(S6) $$\displaystyle \frac{d\overline{N_{I}N_{M}}}{dt}=\left (b_{I}+b_{M}-d_{I}-d_{M}-r\right)\overline{N_{I}N_{M}}+r\overline{N_{I}^{{2}}}-r\overline{N}_{I}$$

#### Analysis of the correlation *C*_size-CD27+_

The correlation *C*_size-CD27+_ describes the correlation between the size or the total population ($N_{I}+N_{M}$) of the NK cell clones and the fraction ($\frac{N_{I}}{N_{I}+N_{M}}$) of the immature CD27+ NK cells at any time *t*. For simplicity of notation, we rename *C*_size-CD27+_ as $Corr\left (N_{I}+N_{M},\frac{N_{I}}{N_{I}+N_{M}}\right)$ for this section. The standard deviation *σ_x_* for a variable *x* is given by $\sigma _{x}=\sqrt{\overline{x^{{2}}}-\overline{x}^{2}}$ ; the overbar represents average over the probability distribution, $P_{N_{I},N_{M}}$

(S7) $$\displaystyle Corr\left (N_{I}+N_{M},\frac{N_{I}}{N_{I}+N_{M}}\right)=\frac{\overline{\left (\left (N_{I}+N_{M}-\overline{\left (N_{I}+N_{M}\right)}\right)\left (\frac{N_{I}}{N_{I}+N_{M}}-\overline{\frac{N_{I}}{N_{I}+N_{M}}}\right)\right)}}{\sigma _{N_{I}\mathrm{+}N_{M}}\sigma _{\frac{N_{I}}{N_{I}+N_{M}}}}$$

We now evaluate conditions that can make the above correlation negative at *t*=8 days. Since the denominator in Equation S7 is positive, we consider the numerator for the analysis. The numerator can be expanded in terms of fluctuations around the mean values of *N*_*I*_ and *N*_*M*_, that is, $N_{I}\left (t\right)=\overline{N_{I}}\left (t\right)+\delta N_{I}\left (t\right)$ and $N_{M}\left (t\right)=\overline{N}_{M}\left (t\right)+\delta N_{M}\left (t\right)$, as follows

(S8) $$\displaystyle =\overline{N}_{I}-\left (\overline{N}_{I}+\overline{N}_{M}\right)\overline{\left (\frac{\frac{\overline{N}_{I}+\delta N_{I}}{\overline{N}_{I}+\overline{N}_{M}}}{{1}+\frac{\delta N_{I}+\delta N_{M}}{\overline{N}_{I}+\overline{N}_{M}}}\right)}$$

Now, considering fluctuations where $\frac{\delta N_{I}+\delta N_{M}}{\overline{N}_{I}+\overline{N}_{M}}{< }1$, we expand the above expression to the second order as

(S9) $$\displaystyle \approx \overline{N}_{I}-\left (\overline{N}_{I}+\overline{N}_{M}\right)\overline{\left (\left (\frac{\overline{N}_{I}+\delta N_{I}}{\overline{N}_{I}+\overline{N}_{M}}\right)\left (1-\frac{\delta N_{I}+\delta N_{M}}{\overline{N}_{I}+\overline{N}_{M}}+\left (\frac{\delta N_{I}+\delta N_{M}}{\overline{N}_{I}+\overline{N}_{M}}\right)^{{2}}\right)\right)}$$

Reducing and noting that $\overline{\delta N_{I}}$ and $\overline{\delta N_{M}}$ are zero,

(S10) $$\displaystyle =\frac{1}{\left (\overline{N}_{I}+\overline{N}_{M}\right)^{2}}\frac{\overline{N}_{M}\delta \overline{N_{I}^{{2}}}+\left (\overline{N}_{M}-\overline{N}_{I}\right)\overline{\delta N_{I}\delta N_{M}}-\overline{N}_{I}\delta \overline{N_{M}^{{2}}}}{\sigma _{\frac{N_{I}}{N_{I}+N_{M}}}\sigma _{N_{I}+N_{M}}}$$

Inputting this in Equation S10,

(S11) $$\displaystyle Corr\left (N_{I}+N_{M},\frac{N_{I}}{N_{I}+N_{M}}\right)\approx \frac{1}{\left (\overline{N}_{I}+\overline{N}_{M}\right)^{2}}\frac{\overline{N}_{M}\sigma _{N_{I}}^{2}+\left (\overline{N}_{M}-\overline{N}_{I}\right)Cov\left (N_{I},N_{M}\right)-\overline{N}_{I}\sigma _{N_{M}}^{2}}{\sigma _{\frac{N_{I}}{N_{I}+N_{M}}}\sigma _{N_{I}+N_{M}}}$$

where

(S12) $$\displaystyle Cov\left (N_{I},N_{M}\right)=\overline{N_{I}N_{M}}-\overline{N}_{I}\overline{N}_{M}$$

Note that $\overline{N}_{M}-\overline{N}_{I}{> }0$ for the NK cell populations at day 8. We checked the validity of the approximation in Equation S11 with our numerical evaluation of the correlation function in Equation S7 (Figure 1—figure supplement 1). We find that the sign of approximate correlation function in Equation S11 agrees with the sign of the actual correlation function in Equation S7. Therefore, use the approximate form in Equation S11 for further analysis. The approximate form of the correlation function in Equation S11 can become negative if $\sigma _{N_{M}}^{2}$ is larger than $\sigma _{N_{I}}^{2}+\left (\overline{N}_{M}-\overline{N}_{I}\right)Cov\left (N_{I},N_{M}\right)$. Below we show that increasing the death rate *d_M_* increases $\sigma _{N_{M}}^{2}$ when the growth rate of the mature population $k_{M}=b_{M}-d_{M}$ and the other rates, *b_I_*, *d_I_*, and *r* are held fixed. Equation S5 can be recast in terms of *k*_M_ and the other rate parameters as

(S13) $$\displaystyle \frac{d\overline{N_{M}^{{2}}}}{dt}=2k_{M}\overline{N_{M}^{2}}+\left (k_{M}+2d_{M}\right)\overline{N}_{M}+2r\overline{N_{I}N_{M}}+r\overline{N}_{I}$$

Since the variables $\overline{N}_{I}$ and $\overline{N}_{M}$ do not depend on $d_{M}$ when Equations S2 and S3 are recast in terms of k_M_, $\frac{\partial \overline{N_{M}^{\mathrm{2}}}}{\partial d_{M}}$ follows the equation below:

(S14) $$\displaystyle \frac{d}{dt}\frac{\partial \overline{N_{M}^{2}}}{\partial d_{M}}=2k_{M}\frac{\partial \overline{N_{M}^{2}}}{\partial d_{M}}+2\overline{N}_{M}$$

where the solution is given by

(S15) $$\displaystyle \frac{\partial \overline{N_{M}^{2}}}{\partial d_{M}}=\frac{\partial \overline{N_{M}^{2}}}{\partial d_{M}}\left (t=0\right)+2e^{2k_{M}t}\int _{0}^{t}dt^{'}e^{-2k_{M}t^{'}}\overline{N}_{M}$$

We can simplify the solution further as

(S16) $$\displaystyle \frac{\partial \overline{N_{M}^{2}}}{\partial d_{M}}=\frac{2r}{k_{M}\left (g-k_{M}\right)\left (g-2k_{M}\right)}\left (k_{M}e^{rt}+\left (g-2k_{M}\right)e^{k_{M}t}-\left (g-k_{M}\right)e^{2k_{M}t}\right)$$

where

$$\displaystyle g=b_{I}-d_{I}-r$$

Equation S16 shows $\frac{\partial \overline{N_{M}^{2}}}{\partial d_{M}}\left (t=0\right)=0$, therefore from Equation S2 $\frac{\partial \overline{N_{M}^{2}}}{\partial d_{M}}\left (t\right)=2\int _{0}^{t}dt^{'}e^{-2k_{M}\left (t^{'}-t\right)}\overline{N}_{M}\left (t'\right)\geq 0$ as the integrand is always non-negative. Since, for the parameters $\overline{N}_{M}$ would be non-zero for t≤8 days, we can conclude that increasing the death rate $d_{M}$ would increase the variance $\sigma _{N_{M}}^{2}$ causing the correlation to become negative.

#### Numerical integration of Master equation and calculation of AIC_c_

We numerically solved the Master equation in Equation S1, which describes the probability distribution $P_{N_{I},N_{M}}\left (t\right){\ }$ of the numbers of immature ($N_{I}$) and mature ($N_{M}$) NK cells at any time *t* given the initial condition $P_{1,0}=1$ at time t=0. We used a numerical integration method to solve the coupled ordinary differential equations (ODEs) in Equation S1. Because there is no upper bound to the number of $N_{I}$ and $N_{M}$, an exact solution of Equation S1 would need to consider the ranges $0\leq N_{I}\leq \infty $ and $0\leq N_{M}\leq \infty .$ However, accounting for the above ranges in a numerical solution of the ODEs will be computationally infeasible; therefore, we approximated the above system of ODEs by considering large but finite upper bounds of $N_{I}$ and $N_{M}$, i.e., $0\leq N_{I}\leq N_{I}^{\left (bound\right)}$ and $0\leq N_{M}\leq N_{M}^{\left (bound\right)}$ where $N_{I}^{\left (bound\right)}$ = $N_{M}^{\left (bound\right)}=1200$. The transition rates carrying the system from inside the boundary to outside the boundary or vice versa were set to zero. This system will provide a good approximation for the full solution for a time interval when the exact solution of the kinetics has a very low probability to transition across the boundary. We checked this with the exact solution for the pure birth model for the time interval of 0–8 days where the largest difference in the probability distributions between the exact and the approximate solutions was ${< }10^{-9}$ (Figure 1—figure supplement 1c). We used ‘*odeint’* function with eighth -order Runge–Kutta method ‘*dopri8*’ from ‘*torchdiffeq’* and implemented those in GPUs to numerically solve the 10^6^ coupled ODEs in Equation S1 with the above bounds in N_I_ and N_M_. We then computed the likelihood *L* of the observed 38 clone sizes ($\left \{N_{I}^{\left (i\right)},N_{M}^{\left (i\right)},i\mathrm{=}1,\dots,38\right \}$) (Figure 1—figure supplement 1b) given the numerical solution of $P\left (N_{I},N_{M};\theta \right)$ , where θ denotes the parameters $b_{I},b_{M},d_{I},d_{M}$ and *r*. The likelihood $L\left (\theta \right)$ is given by

$$\displaystyle L\left (\theta \right)=\prod _{i=1}^{38}P\left (N_{I}^{\left (i\right)},N_{M}^{\left (i\right)};\theta \right)$$

We find optimal parameters ($\hat{\theta}$) by minimizing −log(L(θ)) (Figure 1—figure supplement 1d) using the ‘*minimize’* function with *‘Nelder-Mead’* algorithm from ‘*scipy’*. Then we compute AICc for comparing the models, where *AICc* for a model with *K* parameters describing *n* number of data points is defined as

$$\displaystyle AIC_{c}=AIC+\frac{2K\left (K+1\right)}{n-K-1}$$

and the AIC is given by $AIC=2K-2{\rm log} \left (L\left (\hat{\theta }\right)\right)$.

The ${\rm log} \left (L\left (\hat{\theta }\right)\right)$ values for the two-stage model without death and with death are −545.4 and −514.6, respectively. The corresponding $AICc{\ }$ values are 1097.5 and 1041.0, respectively. Thus, the model with death has a smaller $AICc{\ }$ compared to the model without death with a difference of $\Delta AICc{> }50,$ clearly demonstrating that the model with death is favored over the other model by the observed data.

## Appendix 2

### Moment equations for the three-stage and other models

#### Three-stage model

(S17)
$$\displaystyle \frac{d\overline{N}_{I}}{dt}=\left (b_{I}-d_{I}-r_{1}\right)\overline{N}_{I}$$

(S18)
$$\displaystyle \frac{d\overline{N}_{INT}}{dt} = r_1\overline{N}_I + \left(b_{INT}-d_{INT}-r_2\right)\overline{N}_{INT}$$

(S19)
$$\displaystyle \frac{d\overline{N}_{M}}{dt}=r_{2}\overline{N}_{INT} +\left (b_{M}-d_{M}\right)\overline{N}_{M}$$

(S20)
$$\displaystyle \frac{d\overline{N_{I}^{2}}}{dt}=2\left (b_{I}-d_{I}-r_{1}\right)\overline{N_{I}^{2}}+\left (b_{I}+d_{I}+r_{1}\right)\overline{N}_{I}$$

(S21)
$$\displaystyle \frac{d\overline{N_{INT}^{2}}}{dt} = 2\left(b_{INT}-d_{INT}-r_2\right)\overline{N_{INT}^{2}} + \left(b_{INT}+d_{INT}+r_2\right)\overline{N}_{INT} + 2r_1\overline{N_I N_{INT}} + r_1\overline{N}_I$$

(S22)
$$\displaystyle \frac{d\overline{N_{M}^{2}}}{dt}=2\left (b_{M}-d_{M}\right)\overline{N_{M}^{2}}+\left (b_{M}+d_{M}\right)\overline{N}_{M}+2r_{2}\overline{N_{INT} N_{M}}+r_{2}\overline{N}_{INT}$$

(S23)
$$\displaystyle \frac{d\overline{N_I N_{INT}}}{dt} = \left(b_I+b_{INT}-d_I-d_{INT}-r_1-r_2\right)\overline{N_I N_{INT}} + r_1\overline{N_I^2} - r_1\overline{N}_I$$

(S24)
$$\displaystyle \frac{d\overline{N}_{INT}N_M}{dt} = \left(b_{INT}+b_M-d_{INT}-d_M-r_2\right)\overline{{N}_{INT}N_M} + r_2\overline{N_{INT}^{2}} - r_2\overline{N}_{INT} + r_1\overline{N_I N_M}$$

(S25)
$$\displaystyle \frac{d\overline{N_{I}N_{M}}}{dt}=\left (b_{I}+b_{M}-d_{I}-d_{M}-r_{1}\right)\overline{N_{I}N_{M}}+r_{2}\overline{N_{I}N_{INT}}$$

To change the above system to the model with three stages but the intermediate stage is CD27+, simply replace the parameters *b_INT_* and *d_INT_* with *b_DP_* and *d_DP_*.

#### Two-stage model with asymmetric division

(S26)
$$\displaystyle \frac{d\overline{N}_{I}}{dt}=\left (b_{sym}-d_{I}-r\right)\overline{N}_{I}$$

(S27)
$$\displaystyle \frac{d\overline{N}_{M}}{dt}=\left (r+b_{asym}\right)\overline{N}_{I}+\left (b_{M}-d_{M}\right)\overline{N}_{M}$$

(S28)
$$\displaystyle \frac{d\overline{N_{I}^{2}}}{dt}=2\left (b_{sym}-d_{I}-r\right)\overline{N_{I}^{2}}+\left (b_{sym}+d_{I}+r\right)\overline{N}_{I}$$

(S29)
$$\displaystyle \frac{d\overline{N_{M}^{2}}}{dt}=2\left (b_{M}-d_{M}\right)\overline{N_{M}^{2}}+\left (b_{M}+d_{M}\right)\overline{N}_{M}+2\left (r+b_{asym}\right)\overline{N_{I}N_{M}}+\left (r+b_{asym}\right)\overline{N}_{I}$$

(S30)
$$\displaystyle \frac{d\overline{N_{I}N_{M}}}{dt}=\left (b_{sym}+b_{M}-d_{I}-d_{M}-r\right)\overline{N_{I}N_{M}}+\left (r+b_{asym}\right)\overline{N_{I}^{2}}-r\overline{N}_{I}$$

#### Two-stage model with inflow

(S31)
$$\displaystyle \frac{d\overline{N}_{DP}}{dt}=a+\left (b_{DP}-d_{DP}-r\right)\overline{N}_{DP}$$

(S32)
$$\displaystyle \frac{d\overline{N}_{M}}{dt}=r\overline{N}_{DP}+\left (b_{M}-d_{M}\right)\overline{N}_{M}$$

(S33)
$$\displaystyle \frac{d\overline{N_{DP}^{2}}}{dt} = 2\left(b_{DP}-d_{DP}-r\right)\overline{N_{DP}^{2}} + \left(b_{INT}+d_{INT}+r+2a\right)\overline{N}_{DP} + a$$

(S34)
$$\displaystyle \frac{d\overline{N_{M}^{2}}}{dt}=2\left (b_{M}-d_{M}\right)\overline{N_{M}^{2}}+\left (b_{M}+d_{M}\right)\overline{N}_{M}+2r\overline{N_{DP}N_{M}}+r\overline{N}_{DP}$$

(S35)
$$\displaystyle \frac{d\overline{N_{DP}N_M}}{dt} = \left(b_{INT}+b_M-d_{INT}-d_M-r\right)\overline{N_{DP}N_M} + r\overline{N_{DP}^{2}} - r\overline{N}_{DP} + a\overline{N}_M$$

## Appendix 3

### Additional tables

**Appendix 3—table 1.** Best-fit parameters to the two-stage model with birth and death describing NK cell clonal bursts given in Figure 1e.

Parameter	Estimate
*b*_*I*_	0.662 day^–1^
*b*_*M*_	1.441 day^–1^
*d*_*I*_	0.129 day^–1^
*d*_*M*_	0.964 day^–1^
*r*	0.135 day^–1^

**Appendix 3—table 2.** Best-fit parameters to the asymmetric division model describing NK cell clonal bursts given in Figure 1—figure supplement 4.

Parameter	Estimate (confidence interval)
*b*_sym_	0.716 day^–1^ (0.704–0.811)
*b*_asym_	0.708 day^–1^ (0.663–0.725)
*b*_*M*_	1.406 day^–1^ (1.362–1.442)
*d*_*I*_	0.039 day^–1^ (0.026–0.097)
*d*_*M*_	0.432 day^–1^ (0.401–0.481)
*r*	0.017 day^–1^ (0.004–0.025)

**Appendix 3—table 3.** Best-fit parameters to the three-stage model with two CD27+ stages describing NK cell clonal bursts given in Figure 2—figure supplement 1a.

Parameter	Estimate (confidence interval)
*b*_*I*_	0.899 day^–1^ (0.803–0.963)
*b*_*DP*_	1.450 day^–1^ (1.247–1.801)
*b*_*M*_	0.995 day^–1^ (0.992–2.000)
*d*_*I*_	0.000 day^–1^ (0.000–0.007)
*d*_*DP*_	0.004 day^–1^ (0.000–0.271)
*d*_*M*_	0.076 day^–1^ (0.067–1.055)
*r*_1_	0.017 day^–1^ (0.000–0.018)
r_2_	0.591 day^–1^ (0.418–0.962)

**Appendix 3—table 4.** Best-fit parameters to the two-stage model with constant inflow describing NK cell clonal bursts given in Figure 2—figure supplement 3a.

Parameter	Estimate
*b*_*I*_	1.464 day^–1^
*b*_*M*_	1.596 day^–1^
*d*_*I*_	0.332 day^–1^
*d*_*M*_	0.521 day^–1^
*r*	0.263 day^–1^
*a*	0.886 cell/day

**Appendix 3—table 5.** Best-fit parameters to endogenous NK cells responding to MCMV infection.

Parameter	Estimate (confidence interval)
*k*_*I*_	0.108 day^–1^ (0.034–0.239)
*k*_*INT*_	1.775 day^–1^ (0.018–2.000)
*k*_*M*_	–2.000 day^–1^ (−2.000–0.367)
*r*_1_	3.85×10^–18^ day^–1^ (0.000–0.106)
*r*_2_	1.482 day^–1^ (0.000–1.720)

**Appendix 3—table 6.** Best-fit parameters to the three-stage model with two CD27- stages describing NK cell clonal bursts given in Figure 2a, but while fitting to comparative growth rates of CD27+ vs. CD27- cells rather than constraining to *Δ_k_ >* 0. Because *r*_2_ ≃ 0 and *Δ_k_ <* 0, we report the constrained fit in the main text.

Parameter	Estimate
*b*_*I*_	1.375 day^–1^
*b*_*INT*_	2.000 day^–1^
*b*_*M*_	0.263 day^–1^
*d*_*I*_	0.350 day^–1^
*d*_*INT*_	0.866 day^–1^
*d*_*M*_	2.000 day^–1^
*r*_1_	0.015 day^–1^
*r*_2_	0.000 day^–1^

**Appendix 3—table 7.** Mass cytometry antibody panel.

Metal	Channel	Target
In	113	CD45
In	115	–
La	139	CD62L
Pr	141	Ly6C
Nd	142	CD132
Nd	143	NKR-P1B
Nd	144	CD16
Nd	145	–
Nd	146	CD96
Sm	147	2B4
Nd	148	IFNAR
Sm	149	DNAM-1
Nd	150	CD127
Eu	151	Ly49D
Sm	152	CD49b
Eu	153	CD32
Sm	154	CD69
Gd	155	CD8
Gd	156	CEACAM-1
Gd	157	CD3
Gd	158	NKG2ACE
Tb	159	Ly49H
Gd	160	NK1.1
Dy	161	TIM-3
Dy	162	–
Dy	163	TIGIT
Dy	164	CD2
Ho	165	CD11b
Er	166	CD137
Er	167	CD26
Er	168	Ly49CI
Tm	169	NKp46
Er	170	NKG2D
Yb	171	LAG-3
Yb	172	KLRG1
Yb	173	CD19
Yb	174	Ly49G2
Lu	175	IL18Ra
Yb	176	CD90
Bi	209	CD27
