# Supplementary material for: +Clonal stochasticity in early NK cell response to mouse cytomegalovirus is generated by mature subsets of varying proliferative ability
Source: eLife. 2026 Sep 14;14:RP104951. doi: 10.7554/eLife.104951 (PMC13577665; doi:10.7554/eLife.104951)
Supplement: Peer review history

# Editor report: eLife Assessment

**DOI:** 10.7554/eLife.104951.4.sa0

**Authors:** Frederik Graw

**Affiliations:** Friedrich-Alexander-University Erlangen-Nürnberg Germany

**Keywords:** Solid, Valuable

This study combines mathematical models and experimental data to analyze the emergence of heterogeneity within clonal NK cell responses during antigen-specific cell expansion. It comprises different experimental data and extensively explores various mathematical models, to study NK cell turnover during acute immune responses and homeostatic turnover within murine cytomegalovirus infection (MCMV). This **solid** study presents **valuable** findings and provides relevant insights on heterogeneous NK cell development.

# Referee report: Reviewer #1 (Public review)

**DOI:** 10.7554/eLife.104951.4.sa1

Summary:

The objective of this study was to infer the population dynamics (rates of differentiation, division and loss) and lineage relationships of NK cell subsets during an acute immune response and under homeostatic conditions.

Strengths:

A rich dataset and a detailed analysis of a particular class of stochastic models.

Weaknesses: (relating to initial submission)

The stochastic models used are quite simple; each population is considered homogeneous with first-order rates of division, death, and differentiation. In Markov process models such as these there is no dependence of cellular behavior on its history of divisions. In recent years models of clonal expansion and diversification, in the settings of T and B cells, have progressed beyond this picture. So I was a little surprised that there was no mention of the literature exploring the role of replicative history in differentiation (e.g. Bresser Nat Imm 2022), nor of the notion of family 'division destinies' (either in division number, or the time spent proliferating, as described by the Cyton and Cyton2 models developed by Hodgkin and collaborators; e.g. Heinzel Nat Imm 2017). The emerging view is that variability in clone (family) size arises may arise predominantly from the signals delivered at activation, which dictate each precursor's subsequent degree of expansion, rather than from the fluctuations deriving from division and death modeled as Poisson processes.

As you pointed out, the Gerlach and Buchholz Science papers showed evidence for highly skewed distributions of family sizes, and correlations between family size and phenotypic composition. Is it possible that your observed correlations could arise if the propensity for immature CD27+ cells to differentiate into mature CD27- cells increases with division number? The relative frequency of the two populations would then also be impacted by differences in the division rates of each subset - one would need to explore this. But depending on the dependence of the differentiation rate on division number, there may be parameter regimes (and timepoints) at which the more differentiated cells can predominate within large clones even if they divide more slowly than their immature precursors. One might not then be able to rule out the two-stage model. I would like to see a discussion or rebuttal of these issues.

Comments on revised version.

I am happy with the latest revisions that the authors have made.

# Author comment: Author response

**DOI:** 10.7554/eLife.104951.4.sa2

**Authors:** Darren Wethington, Saeed Ahmad, Marc Potempa, Giuseppe Giuliani, Oscar Aguilar, Maheshwor Poudel, Simon Grassmann, William C Stewart, Nicholas M Adams, Joseph Sun, Lewis Lanier, Jayajit Das

**Affiliations:** Steve and Cindy Rasmussen Institute for Genomic Medicine, Abigail Wexner Research Institute at Nationwide Children's Hospital Columbus United States; The Research Institute at the Nationwide Children's Hospital and the Ohio State University Columbus United States; Department of Microbiology and Immunology and the Parker Institute for Cancer Immunotherapy, University of California, San Francisco San Francisco United States; The Research Institute at the Nationwide Children's Hospital and the Ohio State University Columbus United States; Department of Microbiology and Immunology and the Parker Institute for Cancer Immunotherapy, University of California, San Francisco San Francisco United States; The Research Institute at the Nationwide Children's Hospital and the Ohio State University Columbus United States; 7Immunology Program, Memorial Sloan Kettering Cancer Center New York, NY United States; GIG Consulting, Inc Columbus United States; 9Department of Pathology, New York University Grossman School of Medicine New York, NY United States; Memorial Sloan Kettering Cancer Center New York United States; UCSF San Francisco United States; The Ohio State University Columbus United States

The following is the authors’ response to the previous reviews

**Public Reviews:**

**Reviewer #1 (Public review):**

Summary:

The objective of this study was to infer the population dynamics (rates of differentiation, division and loss) and lineage relationships of NK cell subsets during an acute immune response and under homeostatic conditions.

Strengths:

A rich dataset and a detailed analysis of a particular class of stochastic models.

Weaknesses: (relating to initial submission)

The stochastic models used are quite simple; each population is considered homogeneous with first-order rates of division, death, and differentiation. In Markov process models such as these there is no dependence of cellular behavior on its history of divisions. In recent years models of clonal expansion and diversification, in the settings of T and B cells, have progressed beyond this picture. So I was a little surprised that there was no mention of the literature exploring the role of replicative history in differentiation (e.g. Bresser Nat Imm 2022), nor of the notion of family 'division destinies' (either in division number, or the time spent proliferating, as described by the Cyton and Cyton2 models developed by Hodgkin and collaborators; e.g. Heinzel Nat Imm 2017). The emerging view is that variability in clone (family) size arises may arise predominantly from the signals delivered at activation, which dictate each precursor's subsequent degree of expansion, rather than from the fluctuations deriving from division and death modeled as Poisson processes.

As you pointed out, the Gerlach and Buchholz Science papers showed evidence for highly skewed distributions of family sizes, and correlations between family size and phenotypic composition. Is it possible that your observed correlations could arise if the propensity for immature CD27+ cells to differentiate into mature CD27- cells increases with division number? The relative frequency of the two populations would then also be impacted by differences in the division rates of each subset - one would need to explore this. But depending on the dependence of the differentiation rate on division number, there may be parameter regimes (and timepoints) at which the more differentiated cells can predominate within large clones even if they divide more slowly than their immature precursors. One might not then be able to rule out the two-stage model. I would like to see a discussion or rebuttal of these issues.

Comments on revisions:

(1) The authors have put in a lot of effort to address the reviews and have explored alternative models carefully.

We appreciate the reviewers’ comments.

(2) In the sections relating to homeostasis and the endogenous response, as far as I can tell you are estimating net growth rates (the k parameters) throughout - this is to be expected if you're working with just cell numbers and no information relating to proliferation. In these sections there are many places where you refer to proliferation rates and death rates when I think you just mean net positive or net negative growth rates. It's important to be precise about this even if the language can get a bit repetitive. (These net rates of growth or loss relate to clonal rather than cellular dynamics, which may be worth explaining). Later, you do use data relating to dead cells, which in principle can be used to get independent measures of death rates, but these data were not used in the fitting.

We have modified the main text to address the comment.

(3) There is so much evidence that T and B cell differentiation are often contingent on division that it would be very reasonable to consider it as a possibility for NK cells too. (Differentiation could be asymmetric, as you explored, or simply symmetric with some probability per division). These processes can be cast into simple ODE models but no longer allow you to aggregate division and death rates - so for parameter estimation you need to add measures of proliferation (Ki67 or similar) or death. This may be worth some discussion?

We have modified the main text (lines 242-245) to address the comment.

**Reviewer #2 (Public review):**

Summary:

Wethington et al. investigated the mechanistic principles underlying antigen-specific proliferation and memory formation in mouse natural killer (NK) cells following exposure to mouse cytomegalovirus (MCMV), a phenomenon predominantly associated with CD8+ T cells. Using a stochastic modeling approach, the authors aimed to develop a quantitative model of NK cell clonal dynamics during MCMV infection. Starting from a single immature Ly49+CD27+ NK cell, a two-stage linear model (with a death variant) explained the negative correlation between clone size at 8 dpi and the CD27+ fraction, but failed to reproduce the first and second moments of CD27+ and CD27− NK cell populations at 8 dpi. To address this limitation, the authors added an intermediate maturation state, yielding a three-stage model (CD27+Ly6C− → CD27−Ly6C− → CD27−Ly6C+) that fits the first and second moments under two constraints: CD27+ NK cells proliferate faster than CD27− NK cells, and clone size is negatively correlated with the CD27+ fraction (upper bound of −0.2). The model predicts high proliferation in the intermediate state and high death in mature CD27−Ly6C+ cells, and it was validated using Adams et al. (2021) NK reporter mice tracking CD27+/− populations after tamoxifen, allowing discrimination between bone marrow-derived and pre-existing peripheral NK cells. To test the prediction that mature CD27− NK cells have a higher death rate, the authors measured Ly49H+ NK cell viability in the mouse spleen at different time points post-MCMV infection. Data confirmed lower viability of mature (CD27−) than immature (CD27+) cells during days 4-8 post-infection, and a model variant supported that higher CD27− death increases their proportion in the dead cell compartment. Altogether, the authors propose a three-stage quantitative model of antigen-specific expansion and maturation of naïve Ly49H+ NK cells with the trajectory CD27+Ly6C− (immature) → CD27−Ly6C− (mature I) → CD27−Ly6C+ (mature II), highlighting high proliferation in the mature I state and increased death in the mature II state.

Strengths:

Models explaining correlations and first and second moments, supported by analytical investigations, stochastic simulations, and model selection, identify key processes in antigen-specific NK expansion and maturation. The work distinguishes expansion, contraction, and memory in NK cells from CD8+ T cells and informs NK therapy development.

Weaknesses (relating to initial submission):

The conclusions of this paper are largely supported by the available data. However, a comparative analysis with more recent works in the field would be desirable. Clarifications:

(1) Initial Conditions and Grassmann Data: The Grassmann data is used solely as a constraint, while the simulated values of CD27+/CD27− cells could have been directly fitted to the Grassmann data, which assumes a 1:1 ratio of CD27+/CD27− at t = 0. This would allow an alternative initial condition rather than starting from a single CD27+ cell.

(2) Correlation Coefficients in the Three-Stage Model: Although the parameter scan of the three-stage model (Figure 2) demonstrates the potential for negative correlations between colony size and the fraction of CD27+ cells, the calculated correlation coefficients using the fitted parameter values are not shown. Including these would validate that the fitted parameters lie in the negative-correlation regime.

(3) Viability Dynamics and Adaptive Response: The authors measured the time evolution of CD27+/− dynamics and viability over 30 days post-infection (Figure 4). It would be valuable to test whether the three-stage model can reproduce the adaptive response of CD27− cells to MCMV infection, particularly the observed drop in CD27− viability at 5 dpi and its rebound at 8 dpi. Demonstrating this would test whether the model can simultaneously explain viability dynamics and moment dynamics, and would enable sensitivity analysis of CD27− viability with respect to model parameters.

**Recommendations for the authors:**

**Reviewer #1 (Recommendations for the authors):**

Minor points:

(1) line 175 - Here I think you have only ruled out the two state model with no death, and not the two state model in general?

Edited the sentence to address the comment.

(2) Figures 2 and 5 - the phenotypes (CD27+ Ly6C-, etc.) should be clearly labeled above each cell type. Fig 1 could be improved in the same way.

Done.
